# Patient Preference and Risk Assessment in Opioid Prescribing Disparities: A Secondary Analysis of a Randomized Clinical Trial

**DOI:** 10.1001/jamanetworkopen.2021.18801

**Published:** 2021-07-29

**Authors:** Eden Engel-Rebitzer, Abby R. Dolan, Shoshana V. Aronowitz, Frances S. Shofer, Max Jordan Nguemeni Tiako, Marilyn M. Schapira, Jeanmarie Perrone, Erik P. Hess, Karin V. Rhodes, Venkatesh R. Bellamkonda, Carolyn C. Cannuscio, Erica Goldberg, Jeffrey Bell, Melissa A. Rodgers, Michael Zyla, Lance B. Becker, Sharon McCollum, Zachary F. Meisel

**Affiliations:** 1 Center for Emergency Care Policy and Research, University of Pennsylvania, Philadelphia; 2 Department of Emergency Medicine, Perelman School of Medicine, University of Pennsylvania, Philadelphia; 3 National Clinician Scholars Program, University of Pennsylvania, Philadelphia; 4 Yale School of Medicine, New Haven, Connecticut; 5 Leonard Davis Institute of Health Economics, University of Pennsylvania, Philadelphia; 6 Department of General and Internal Medicine, University of Pennsylvania, Philadelphia; 7 Department of Emergency Medicine, Penn Center for Addiction Medicine and Policy, Philadelphia, Pennsylvania; 8 Department of Emergency Medicine, Mayo Clinic College of Medicine and Science, Rochester, Minnesota; 9 Department of Emergency Medicine, Vanderbilt University School of Medicine, Nashville, Tennessee; 10 Department of Emergency Medicine, Donald and Barbara Zucker School of Medicine at Hofstra/Northwell, Hempstead, New York; 11 Center for Public Health Initiatives, University of Pennsylvania, Philadelphia; 12 College of Education, University of Texas at Austin, Austin; 13 Penn Injury Science Center, University of Pennsylvania, Philadelphia

## Abstract

**Importance:**

Although racial disparities in acute pain control are well established, the role of patient analgesic preference and the factors associated with these disparities remain unclear.

**Objective:**

To characterize racial disparities in opioid prescribing for acute pain after accounting for patient preference and to test the hypothesis that racial disparities may be mitigated by giving clinicians additional information about their patients’ treatment preferences and risk of opioid misuse.

**Design, Setting, and Participants:**

This study is a secondary analysis of data collected from Life STORRIED (Life Stories for Opioid Risk Reduction in the ED), a multicenter randomized clinical trial conducted between June 2017 and August 2019 in the emergency departments (EDs) of 4 academic medical centers. Participants included 1302 patients aged 18 to 70 years who presented to the ED with ureter colic or musculoskeletal back and/or neck pain.

**Interventions:**

The treatment arm was randomized to receive a patient-facing intervention (not examined in this secondary analysis) and a clinician-facing intervention that consisted of a form containing information about each patient’s analgesic treatment preference and risk of opioid misuse.

**Main Outcomes and Measures:**

Concordance between patient preference for opioid-containing treatment (assessed before ED discharge) and receipt of an opioid prescription at ED discharge.

**Results:**

Among 1302 participants in the Life STORRIED clinical trial, 1012 patients had complete demographic and treatment preference data available and were included in this secondary analysis. Of those, 563 patients (55.6%) self-identified as female, with a mean (SD) age of 40.8 (14.1) years. A total of 455 patients (45.0%) identified as White, 384 patients (37.9%) identified as Black, and 173 patients (17.1%) identified as other races. After controlling for demographic characteristics and clinical features, Black patients had lower odds than White patients of receiving a prescription for opioid medication at ED discharge (odds ratio [OR], 0.42; 95% CI, 0.27-0.65). When patients who did and did not prefer opioids were considered separately, Black patients continued to have lower odds of being discharged with a prescription for opioids compared with White patients (among those who preferred opioids: OR, 0.43 [95% CI, 0.24-0.77]; among those who did not prefer opioids: OR, 0.45 [95% CI, 0.23-0.89]). These disparities were not eliminated in the treatment arm, in which clinicians were given additional data about their patients’ treatment preferences and risk of opioid misuse.

**Conclusions and Relevance:**

In this secondary analysis of data from a randomized clinical trial, Black patients received different acute pain management than White patients after patient preference was accounted for. These disparities remained after clinicians were given additional patient-level data, suggesting that a lack of patient information may not be associated with opioid prescribing disparities.

**Trial Registration:**

ClinicalTrials.gov Identifier: NCT03134092

## Key Points

**Question:**

What is the role of patient preference in racial disparities in opioid prescribing for patients with acute pain, and does providing clinicians with additional data about their patients mitigate disparities?

**Findings:**

In this secondary analysis of 1012 patients with acute pain who were recruited for a multicenter randomized clinical trial, Black patients were less likely than White patients to receive a prescription for opioids, regardless of their treatment preference. These disparities were not mitigated by providing clinicians with additional data about their patients’ preferences and risks.

**Meaning:**

This study’s findings suggest that differences in patient preference do not explain racial disparities in opioid prescribing; further research is needed to assess the factors associated with these disparities.

## Teaser

This secondary analysis of data from a randomized clinical trial characterizes racial disparities in opioid prescribing and examines whether a clinician intervention describing patient treatment preference and risk of opioid misuse was able to mitigate disparities among patients with acute pain.

## Introduction

The opioid overdose epidemic^1^ has led to increased scrutiny of opioid prescribing in acute care settings, such as the emergency department (ED).^2,3^ Despite efforts to standardize opioid prescribing, racial and ethnic disparities remain in the care of patients with acute pain.^4,5^ Black patients are less likely than White patients to receive opioid medication for acute pain when they are treated in the ED and are more likely to have their pain undertreated.^4,6,7,8^ This disparity has been identified across multiple clinical presentations (eg, musculoskeletal pain, long-bone fractures, and appendicitis) and patient populations (eg, adult vs pediatric).^4,9,10,11^ Although opioid medications may or may not be indicated for the treatment of acute pain, these findings are concerning because racial differences in pain management are inherently problematic.^12^

The Centers for Disease Control and Prevention recommends that clinicians make acute pain management decisions using a shared decision-making model that weighs patient desires and risks with regard to pain management.^13^ Despite these recommendations, the role of patient preference continues to challenge descriptive studies of racial disparities in opioid prescribing. The possibility that differences in patient preference are associated with clinicians’ prescribing disparities has been proposed but not sufficiently evaluated in the existing literature, much of which consists of cross-sectional studies without access to patient preference data.^14,15,16^ Accounting for preference is especially important in acute pain management because there is no criterion standard treatment, and prescribing decisions are often informed by a discussion between the patient and clinician.^7^

Although racial disparities in the prescribing of pain treatment have been well documented, the factors associated with these disparities are not completely understood, and a wide range of theories has been explored.^17,18,19,20,21,22^ One theory, which has not yet been examined with regard to opioid prescribing disparities, is known as statistical discrimination.^23,24^ According to this theory, when decision-makers make choices with incomplete information, they may generate disparities by applying generalizations about a group to individual members of that group. Inaccurate statistical discrimination is a specific type of statistical discrimination that occurs when decision-makers apply false beliefs about group differences to individuals in an effort to fill in information gaps.^25^ For example, in the case of opioid prescribing, clinicians may apply the false belief that Black patients are at increased risk for opioid use disorder to an individual patient when they are unsure of that patient’s risk.^26^ Disparities generated by inaccurate statistical discrimination may be mitigated by providing clinicians with additional data about their patients. This theory may have particular relevance in the ED, where clinicians must make decisions about patients with whom they are unfamiliar.

The present study addressed these gaps in the literature by examining racial disparities in opioid prescribing within the novel context of a prospective randomized clinical trial (Life Stories for Opioid Risk Reduction in the ED [Life STORRIED]) in which pain management preference was considered. We also tested the hypothesis that, consistent with a model of inaccurate statistical discrimination, we would find a decrease in prescribing disparities when clinicians were given additional data about their patients’ opioid-associated risks and preferences.

## Methods

### Study Design and Setting

The present study is a secondary analysis of outcomes (not prespecified) using data collected from the Life STORRIED randomized clinical trial.^27^ The clinical trial was conducted in the EDs of 4 academic medical centers: the University of Pennsylvania (Philadelphia), Northwell Health (Manhasset, New York), the Mayo Clinic (Rochester, Minnesota), and the University of Alabama (Birmingham). The parent study, including secondary analyses, was approved by the University of Pennsylvania Institutional Review Board. All participants provided written informed consent for both primary and additional analyses. The study followed the Consolidated Standards of Reporting Trials (CONSORT) reporting guideline for parallel randomized clinical trials (eFigure in Supplement 2).^28^

### Participants

Participants were enrolled between June 2017 and August 2019. Inclusion criteria comprised (1) presentation to the ED for uncomplicated ureter colic or musculoskeletal back and/or neck pain, (2) age 18 to 70 years, (3) ability to comprehend English, and (4) clinician intention to discharge the patient within 24 hours of enrollment. Exclusion criteria included any contraindication to opioid or nonsteroidal anti-inflammatory drugs and the use of opioid medication in the 30 days before ED presentation.^27^ Trained research associates (A.D. and E.G.) identified eligible participants based on a review of the electronic medical record and a conversation with the participant’s ED clinician.

### Study Protocol

Research associates enrolled participants and obtained written informed consent. Randomization occurred automatically through Way to Health,^29^ a web-based data collection platform for behaviorally oriented clinical trials. After randomization, patients completed a series of surveys, including a demographic survey, a pain management preference survey, and the Opioid Risk Tool (ORT), a survey validated for assessing the risk of opioid misuse (score range, 0-26, with 0-3 indicating low risk, 4-7 indicating moderate risk, and ≥8 indicating high risk).^30^ The clinical trial was closed at completion of recruitment (Trial Protocol available in Supplement 1)

### Intervention

The Life STORRIED clinical trial comprised 3 arms: a probabilistic risk tool (PRT) arm, a narrative-enhanced probabilistic risk tool (NE-PRT) arm, and a general risk information sheet (control) arm. The experimental arms (PRT and NE-PRT) included a patient-facing and clinician-facing intervention, both of which were administered during the ED visit. The patient-facing intervention in the PRT arm consisted of a tablet application based on the ORT that provided each patient with their individualized risk of opioid misuse.^30^ Research associates were trained to administer the ORT using nonstigmatizing language. Patients in the NE-PRT arm received the same intervention as those in the PRT arm but were also exposed to an additional narrative intervention that consisted of video vignettes showing real patients speaking about their experiences with acute pain and opioid medications.^27^

**Figure 1.**
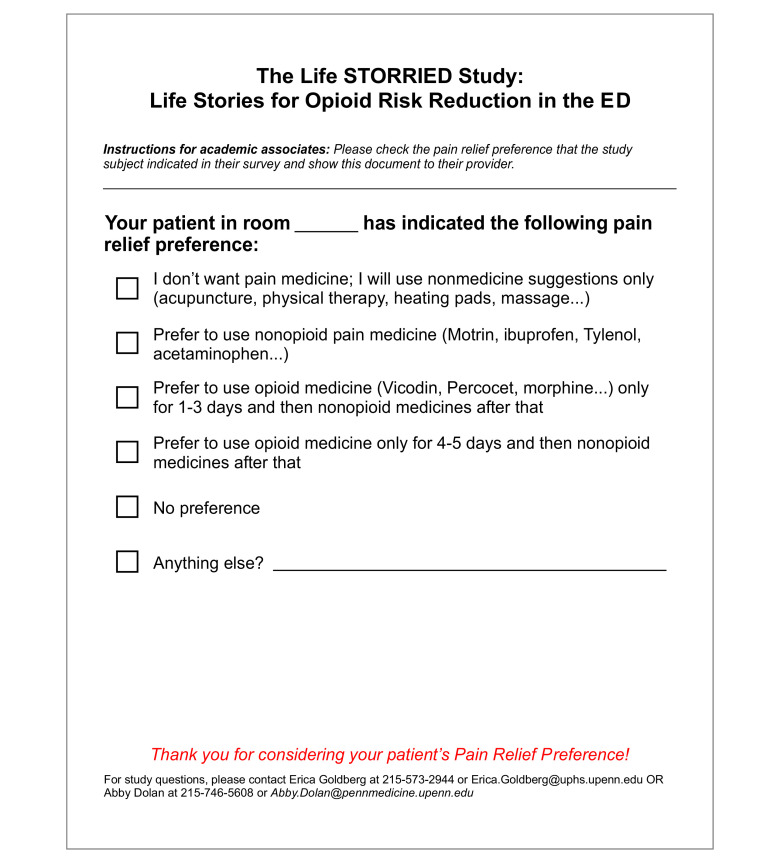
Patient Preference Communication Form ED indicates Emergency Department; Life STORRIED, Life Stories for Opioid Risk Reduction in the ED.

**Figure 2.**
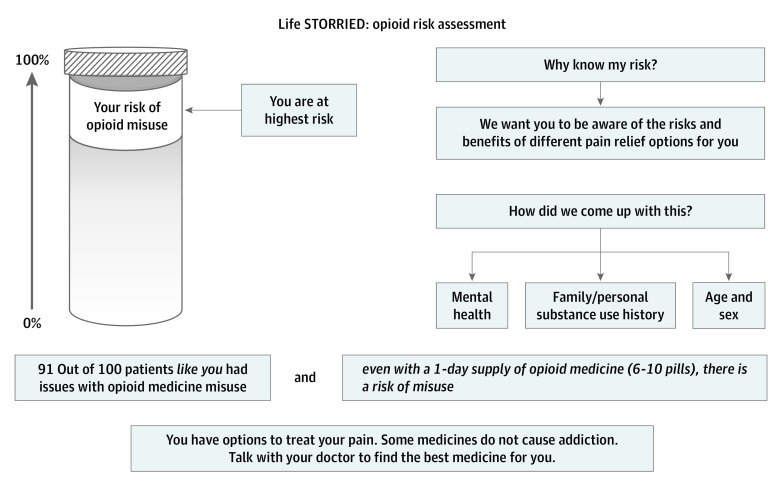
Opioid Misuse Risk Communication Form Life STORRIED indicates Life Stories for Opioid Risk Reduction in the ED.

In addition to the patient-facing interventions, ED clinicians who treated patients in the PRT and NE-PRT arms were exposed to an additional clinician-facing intervention. The clinician-facing intervention consisted of a prepopulated form describing the patient’s treatment preference and risk of opioid misuse (Figure 1 and Figure 2). For the purposes of this secondary analysis, the impact of the clinician-facing intervention was examined by combining the PRT and NE-PRT arms into a single treatment arm.

### Measures

We assessed the risk of opioid misuse via the ORT. We collected self-reported data about patient race, educational level, age, and self-identified gender via a demographic survey administered during the ED visit. Participants who identified as neither White nor Black were included in a third category, which was referred to as other race. This group included individuals identifying as American Indian, Asian, and Pacific Islander as well as those who reported multiple races and other races. We defined severe pretreatment pain as pain that patients reported to be 7 or higher on a 10-point validated verbal numeric rating scale.^31^

Trained research associates abstracted ED visit data, including pretreatment pain level (assessed during ED triage) and the presence or absence of an opioid prescription at ED discharge, from the electronic medical record. We assessed treatment preference via a survey question that offered five treatment modalities for patients to select: (1) no pain medication, (2) nonopioid pain medication, (3) opioid medication for 1 to 3 days followed by nonopioid medication, (4) opioid medication for 4 to 5 days followed by nonopioid medication, and (5) no preference. Participants who endorsed a preference for the receipt of opioid medication for any length of time were classified as preferring opioids. Participants who reported no preference were excluded from the analysis, and all other patients were classified as not preferring opioids.

This study had 2 primary outcome measures: (1) patient receipt of an opioid prescription at ED discharge and (2) concordance between preferred and received treatment. Treatment was defined as concordant if a participant who preferred opioids received a prescription for opioids at discharge or if a participant who did not prefer opioids did not receive a prescription for opioids at discharge. To allow a more granular examination of discordance, we also examined opioid prescriptions separately among patients who did and did not prefer opioids.

### Statistical Analysis

Two-sided χ^2^ tests of independence were conducted to compare demographic characteristics between the treatment and control arms. These χ^2^ tests were also used to examine the associations between race and ORT risk category and between race and our outcome variables. Multivariable logistic regression models were used to examine the association between race and each outcome variable. We controlled for assignment to the NE-PRT arm of the study to account for potential differences associated with the additional patient-facing intervention in that group. To examine whether the clinician-facing intervention moderated the association between race and our outcome variables, we included an interaction term between race and treatment arm in our models. We set *P* < .05 as the significance threshold. All analyses were performed using Stata statistical software, version 16.1 (StataCorp LLC).

## Results

Among 1302 patients enrolled in the Life STORRIED clinical trial, 1301 patients were randomized; 1012 patients with complete data on demographic characteristics and treatment preference were included in this secondary analysis. Of those, 671 patients (66.3%) were assigned to the treatment arm, and 341 patients (33.7%) were assigned to the control arm. A total of 563 patients (55.6%) self-identified as female, 446 patients (44.1%) self-identified as male, and 3 patients (0.3%) self-identified as neither male nor female; the mean (SD) age was 40.8 (14.1) years. Most of the sample (766 patients [75.7%]) presented to the ED with back and/or neck pain. A total of 455 patients (45.0%) identified as White, 384 patients (37.9%) identified as Black, and 173 patients (17.1%) identified as other races. No significant differences were observed between the control arm and the combined treatment arm with regard to self-identified race, self-identified gender, age, or presenting condition (Table 1).

**Table 1.** Patient Demographic Characteristics

Characteristic	No. (%)	
	Control arm	Treatment arm
Total patients, No.	341	671
Age, mean (SD), y^a^	40.2 (14.0)	41.1 (14.2)
Self-identified gender		
Male	157 (46.0)	289 (43.1)
Female	184 (54.0)	379 (56.5)
Other	0	3 (0.4)
Self-identified race		
Black	137 (40.2)	247 (36.8)
White	150 (44.0)	305 (45.5)
Other^b^	54 (15.8)	119 (17.7)
Condition		
Ureter colic	76 (22.3)	170 (25.3)
Back and/or neck pain	265 (77.7)	501 (74.7)

^a^ A total of 1010 patients were included in the age analysis (2 participants were missing data on age).

^b^ Other races included individuals who self-identified as American Indian, Asian, and Pacific Islander as well as those who reported multiple races or other race.

Among 1010 participants with complete data on treatment preference and opioid prescription, 377 patients (37.3%) reported a preference for opioids at ED discharge, 238 patients (23.6%) received a prescription for opioids at ED discharge, and 659 patients (65.2%) received treatment concordant with their preference. Most of the sample (763 of 1012 patients [75.4%]) had ORT scores that corresponded to a low risk of opioid misuse. No differences in ORT risk category were found between Black and White patients; however, other racial groups were more likely to be at low risk than both Black and White patients (145 patients [83.8%] of other races vs 281 Black patients [73.2%; *P* = .02] vs 337 White patients [74.1%; *P* = .03]). No significant differences in treatment preference were observed between the 3 racial groups.

White patients were more likely to receive a prescription for opioids at ED discharge compared with both Black patients and patients in other racial groups (153 of 454 White patients [33.7%] vs 47 of 384 Black patients [12.2%] vs 38 of 172 patients [22.1%] of other races; *P* < .001) (Table 2). After controlling for demographic characteristics and clinical features (including pretreatment pain), the odds of Black patients (odds ratio [OR], 0.42; 95% CI, 0.27-0.65) and patients in other racial groups (OR, 0.59; 95% CI, 0.37-0.94) receiving an opioid prescription were lower than those of White patients (Table 3). When we adjusted for recruitment site fixed effects, Black patients remained significantly less likely to receive a prescription for opioids than White patients (OR, 0.59; 95% CI, 0.36-0.97) (eTable 1 in Supplement 2). No significant interaction was observed between race and treatment arm (eTable 2 in Supplement 2).

**Table 2.** Concordance and Discordance Outcomes by Race

Preference	Prescribed opioids	Patients, No. (%)			
		All (n = 1010)^a^	Black race (n = 384)	White race (n = 454)	Other race (n = 172)^b^
Preferred opioids	Yes	132 (13.1)	30 (7.8)	83 (18.3)	19 (11.0)
	No	245 (24.3)	115 (29.9)	93 (20.5)	37 (21.5)
Did not prefer opioids	Yes	106 (10.5)	17 (4.4)	70 (15.4)	19 (11.0)
	No	527 (52.2)	222 (57.8)	208 (45.8)	97 (56.4)

^a^ A total of 1010 of 1012 patients were included in this analysis because 2 patients were missing data on preference or opioid prescription.

^b^ Other races included individuals who self-identified as American Indian, Asian, and Pacific Islander as well as those who reported multiple races or other race.

**Table 3.** Logistic Regression Models With Demographic and Clinical Covariates^a^

Variable	Received an opioid prescription, OR (95% CI)		
	All patients (n = 968)	Patients preferring opioids (n = 364)	Patients preferring no opioids (n = 602)
Self-identified race			
White	1 [Reference]	1 [Reference]	1 [Reference]
Black	0.42 (0.27-0.65)	0.43 (0.24-0.77)	0.45 (0.23-0.89)
Other^b^	0.59 (0.37-0.94)	0.68 (0.34-1.39)	0.62 (0.33-1.19)
Condition			
Ureter colic	1 [Reference]	1 [Reference]	1 [Reference]
Back and/or neck pain	0.18 (0.12-0.26)	0.20 (0.11-0.35)	0.16 (0.09-0.27)
Age	1.00 (0.99-1.01)	1.01 (0.99-1.03)	1.00 (0.99-1.02)
Self-identified gender			
Female	1 [Reference]	1 [Reference]	1 [Reference]
Male	1.56 (1.11-2.18)	1.27 (0.78-2.08)	1.50 (0.91-2.47)
Other	2.27 (0.16-32.02)	1.66 (0.09-31.6)	NA
Educational level			
<High school	1 [Reference]	1 [Reference]	1 [Reference]
High school or some college	0.62 (0.30-1.25)	0.56 (0.23-1.34)	0.75 (0.19-2.90)
≥College	0.63 (0.30-1.32)	0.40 (0.15-1.09)	1.26 (0.32-5.02)
Baseline pain level	1.21 (1.12-1.31)	1.11 (0.98-1.25)	1.23 (1.10-1.38)
ORT score	0.96 (0.91-1.01)	0.96 (0.90-1.02)	0.92 (0.84-1.02)
Narrative arm	1.08 (0.75-1.54)	1.12 (0.66-1.90)	1.19 (0.71-1.97)

Abbreviations: NA, not applicable; OR, odds ratio; ORT, Opioid Risk Tool.

^a^ Sample size varied for these analyses because of missing data on demographic characteristics, discharge prescription, and treatment preference.

^b^ Other races included individuals who self-identified as American Indian, Asian, and Pacific Islander as well as those who reported multiple races or other race.

No significant differences were found between overall preference and treatment concordance by race (291 of 454 White patients [64.1%] received concordant treatment vs 252 of 384 Black patients [65.6%] vs 116 of 172 patients [67.4%] of other races; *P* = .72). Among patients whose treatment preference and prescription receipt were discordant, Black and White patients’ discordance went in different directions. Among 384 Black patients, the largest discordant group was patients who preferred opioids but did not receive prescriptions for opioids at discharge (115 patients [29.9%]); only 17 Black patients (4.4%) did not prefer opioids but received prescriptions for them. Among 454 White patients, the 2 kinds of discordance occurred at similar rates; 70 White patients (15.4%) did not prefer opioids but received prescriptions for them, and 93 White patients (20.5%) preferred opioids but did not receive prescriptions for them (Table 2).

Among 633 patients who did not prefer opioids, White patients were more likely than Black patients and patients of other races to receive prescriptions for opioids (70 White patients [25.2%] vs 17 Black patients [7.1%] vs 19 patients [16.4%] of other races; *P* < .001). Among 227 patients with nonsevere pain who did not prefer opioids, there was no significant difference in opioid prescribing by race (15 White patients [14.9%] vs 4 Black patients [5.0%] vs 3 patients [6.5%] of other races; *P* = .06). After adjusting for demographic characteristics and clinical features, Black patients who did not prefer opioids were less likely than similar White patients to receive an opioid prescription (OR, 0.45; 95% CI, 0.23-0.89) (Table 3). This association was no longer significant after accounting for site of care (eTable 1 in Supplement 2). Assignment to the treatment arm moderated the association between race and opioid prescription. When we estimated our regression model separately for each study arm, Black patients in the treatment arm had lower odds of receiving a prescription for opioids than White patients in the treatment arm, despite not preferring opioids (OR, 0.32; 95% CI, 0.18-0.55); this association between race and receiving a prescription for opioids was not observed in the control arm.

Among 377 patients who reported a preference for opioids, White patients were more likely than Black patients to be discharged from the ED with an opioid prescription (83 White patients [47.2%] vs 30 Black patients [20.7%] vs 19 patients [33.9%] of other races; *P* < .001). Among 280 patients with severe pain who preferred opioids, Black patients remained less likely than White patients and patients of other races to receive an opioid prescription at ED discharge (24 Black patients [22.0%] vs 60 White patients [47.2%] vs 16 patients [36.4%] of other races; *P* < .001). After adjustment for demographic characteristics and clinical features (364 observations), Black patients who reported a preference for opioids had lower odds than White patients of being discharged with a prescription for opioids (OR, 0.43; 95% CI, 0.24-0.77) (Table 3). This lower likelihood was not observed after recruitment site was adjusted for (OR, 0.50; 95% CI, 0.25-1.00) (eTable 1 in Supplement 2). The association between race and opioid prescription was not moderated by the experimental intervention (eTable 2 in Supplement 2).

## Discussion

This secondary analysis of data from the Life STORRIED clinical trial investigated the role of patient preferences for pain management and statistical discrimination in opioid prescribing disparities within the context of a large multicenter clinical trial. Similar overall rates of concordance between patient treatment preference and treatment received were observed between Black and White patients; however, the type of discordance differed between the 2 groups. Black patients who reported a preference for opioids were less likely than similar White patients to be discharged with an opioid prescription. White patients who did not prefer opioids were more likely than similar Black patients to be discharged with an opioid prescription. These disparities did not decrease when clinicians were given additional patient-level information (ORT risk score and treatment preference).

Among those who did not prefer opioids, White patients were more likely than Black patients to receive a prescription for opioids in the treatment arm but not in the control arm. This disparity may have occurred because most patients in our sample had low ORT scores, and clinicians may have applied this information differently in their encounters with Black and White patients. In other words, clinicians may have been more willing to incorporate a patient’s low ORT risk score into their decision-making when interacting with White vs Black patients. This theory is consistent with the results of previous studies, which have found that clinicians make different pain management decisions in response to identical patient-level information when treating Black vs White patients.^18,32^

This study’s intervention was not associated with a decrease in racial disparities in opioid prescribing. A previous study^25^ found that providing decision-makers (in this case, clinicians) with factual information was associated with mitigation of the disparities generated from inaccurate statistical discrimination. The findings of the present analysis suggest that inaccurate statistical discrimination, at least with regard to patient preference and risk of opioid misuse, may not explain the disparities identified in this analysis. Other theories proposed in the literature may better explain these findings. For example, cognitive load theory posits that when clinicians have a high cognitive burden (eg, multiple competing tasks or demands), they may rely on false racial stereotypes to make decisions.^33,34^ According to this theory, constraints on clinicians’ time and attention are associated with cognitive load. Therefore, disparities may be mitigated by giving clinicians more distraction-free time, not more data, to make decisions.^19,35,36,37^

Other studies^38,39^ have suggested that disparities in analgesic prescribing may instead be associated with clinician underestimation of, or lack of empathy for, the pain of Black patients. Experimental work has found that observers demonstrate lower physiological arousal in response to pain experienced by Black vs White participants, suggesting that observers may be experiencing less empathy for Black individuals.^40^ Consistent with the theory that disparities in empathy play a role in prescribing disparities, a previous study^41^ also found that exposure to an empathy-inducing intervention may be associated with decreases in opioid prescribing disparities in an experimental setting. In addition, it may be that statistical discrimination is associated with prescribing disparities but that clinicians are using false generalizations about variables other than patient preference and risk of opioid misuse. It is also possible that the present study’s intervention was unsuccessful because clinicians make their prescribing decisions early in a patient’s ED course and are reluctant to change these decisions in response to new information.

The present analysis offers multiple additions to the existing research on opioid prescribing disparities. The use of patient preference allowed us to examine prescribing disparities within a novel shared decision–making framework, in which preferences were both assessed and communicated to clinicians. Unlike previous observational studies, these data, which included information obtained via a well-documented survey, were collected within the context of a prospective clinical trial in a clearly defined population. Therefore, these findings counter the theory that racial disparities are associated with differences in patient-level factors rather than clinician or systemic bias.^14^ To our knowledge, this study is also the first to assess whether sharing additional patient data with clinicians mitigates opioid prescribing disparities.

### Strengths and Limitations

This study has several strengths. These include the enrollment of patients presenting to the ED, a clinical setting in which patients are randomly assigned to clinicians. The inclusion of this sample allowed us to approximate random assignment of patients to clinicians. In addition, we excluded patients who were receiving chronic opioids from the sample. This exclusion is an important strength of the study because patients with chronic pain (eg, those with cancer) may be subject to different opioid prescribing guidelines. Excluding this population strengthened the supposition that differences in opioid prescribing may reflect a true disparity associated with patient race rather than clinical condition or severity of illness.

The study also has limitations. Although all patients were enrolled before ED discharge, it is possible that, in some cases, clinicians were not notified of the patient’s treatment preference and ORT score until after they wrote the patient’s discharge prescription. Based on an audit of the research staff, who enrolled most of the participants, this phenomenon occurred in a small number of cases. Future research is warranted to address this limitation by examining whether giving clinicians more complete patient-level data earlier in the ED course may provide a more successful intervention. Although participants had the option of selecting multiple races, the 51 patients who selected this option were included in the other-race group of our final race variable, even if their clinicians may have perceived them as having Black or White ancestry. In addition, although this study found a disparity in clinician medication selection, the analyses did not explore disparities in patient pain control, which is an important area for future research. Future interventions can promote safe opioid prescribing while ensuring that the needs of patients of different races are equally addressed.

## Conclusions

This study found that Black patients in the ED were less likely than White patients to receive opioid prescriptions for acute pain but were equally likely to receive their desired choice of pain management medication. Among those who did not receive their preferred treatment, White patients received a prescription for opioids more often than they preferred, and Black patients received a prescription for opioids less often than they preferred. These disparities were not eliminated by assessing and providing clinicians with patients’ treatment preferences and risk of opioid misuse. Future research is warranted to further examine alternative factors associated with prescribing disparities.

## Notes

Supplement 1. Trial Protocol

Supplement 2. **eTable 1.** Logistic Regression Models Including Site Fixed Effects **eTable 2.** Logistic Regression Models Including Interaction Between Race and Study Arm **eFigure.** CONSORT Flow Diagram

Supplement 3. Data Sharing Statement
