# Supplementary material for: Patient Preference and Risk Assessment in Opioid Prescribing Disparities: A Secondary Analysis of a Randomized Clinical Trial
Source: JAMA Netw Open. 2021 Jul 29;4(7):e2118801. doi: 10.1001/jamanetworkopen.2021.18801 (PMC8322998; doi:10.1001/jamanetworkopen.2021.18801)
Supplement: Supplement 1. — Trial Protocol [file jamanetwopen-e2118801-s001.pdf]

PRINCIPAL INVESTIGATOR: Dr. Zachary Meisel

PROTOCOL TITLE: The Life STORRIED study: Life Stories for Opioid Risk Reduction in the ED

INTRODUCTION AND PURPOSE:

OBJECTIVES:
The objective of this study is to compare the effectiveness of (A) a standardized general risk information sheet only, (B) a standardized general risk information sheet plus a probabilistic risk tool, (C) standardized sheet plus narrative enhanced probabilistic risk tool on a variety of patient reported and patient centered outcomes related to pain treatment and the risks and benefits of opioid prescriptions for common, acute pain conditions. These objectives will be achieved in a multicenter randomized practical clinical trial of 1,300 patients across 4 sites that are planned for discharge from acute care settings after being treated for acute kidney stone pain (renal colic) or musculoskeletal back pain. We hypothesize that, compared to patients receiving a generalized risk information sheet or a probabilistic risk communication tool alone, patients with acute renal colic and musculoskeletal back pain randomized to receive narrative enhanced risk communication, will: (**H1**) demonstrate greater knowledge as determined by awareness of risk for opioid dependency; (**H2a**) select a treatment plan with fewer opioids; (**H2b**) take fewer opioids for fewer days, while achieving the same degree of pain relief and improved functional status; and (**H3a**) enjoy greater levels of concordance between the patient-preferred and provider-selected treatment plans and (**H3b**) engage in greater shared decision making with their provider.

BACKGROUND:

Impact of the condition on the health of individuals and populations:

The U.S. is experiencing an epidemic of prescription drug abuse, according to the Center for Disease Control and Prevention (CDC), with deaths from poisonings now exceeding that from motor vehicle crashes.(2,3)In 2014 there were nearly 19,000 U.S. overdose deaths associated with prescription opioids.(4) ED visits for nonmedical use of opioid analgesics increased 153% from 2004 to 2011.(5) A recent national report showed that between 1999 and 2010, deaths from prescription opioids surpassed those from heroin and cocaine combined, with misuse and abuse of prescription analgesics totaling an estimated $53.4 billion a year in lost productivity, medical costs and criminal justice costs.(6) This epidemic has been driven by dramatic increases in opioid sales and prescribing by health care providers, resulting in their expanded availability and frequent diversion for nonmedical use.(4,7,8)

Acute Care Settings and the Opioid Epidemic:

Emergency medicine providers (EPs) care for victims of opioid overdose, abuse, and misuse every day. Paradoxically, EPs are also among the top 5 prescribers of opioid medication for patients under age 40 years.(9) With 42% of ED visits related to pain - combined with provider quality measures that include adequacy of pain treatment and patient satisfaction - there has been tremendous pressure for EPs to prescribe opioids to their patients.10 As safety net providers for a vulnerable population without primary care access or continuity of care, EPs (including short-stay observation unit and urgent care providers) have embraced the responsibility for bridging patients from acute pain episodes to follow up care, including providing pain medications when patients cannot access primary care for treatment of pain. In this context, acute care settings are vital locations where providers and their patients must navigate the line between addressing pain and preventing the misuse of opioid pain medication. Prevention, particularly for at risk patients, may be the key to addressing the epidemic, as once

addiction occurs, only one in (10) Americans receives treatment (11) and current treatment approaches show very low rates of success.(12)

Racial and Ethnic Disparities in Pain Treatment Under-treatment of pain (oligoanalgesia) is also common in acute care settings. (13) Causes of under-treatment of pain in the ED include a failure to acknowledge pain,(14,15) to assess and/or document pain,(15) to ask about treatment adequacy and to meet patient expectations of quick adequate pain relief.(16,17) Under-treatment of pain can have serious consequences. In addition to suffering, patients without adequate pain relief are more likely to seek illegal methods of pain control, to abuse opioids once given them, (18) have greater functional limitations, decreased quality of life, impaired sleep, and decreased immune function, cognition, and mobility.(19) Women, minorities, and the elderly are most likely to be under-treated for pain. (19,21) Minority populations also have a higher risk of experiencing severe pain, having more functional impairment as a result of pain, and a poorer quality of life related to pain.(22)

Opioid Prescriptions for Acute Pain:

A recent study found that approximately 8% of opioid naïve patients prescribed opioids within seven days of short-stay surgery were still taking opioids one year later.(23) The risk of long term opioid use after a first prescription for acute pain from the ED has been specifically studied: Hoppe and colleagues demonstrated that among opioid naïve patients receiving an ED opioid prescription, 12% had more opioids prescribed at one year. Among young adults, a single legitimate prescribed opioid in high school was associated with a 33% increase in the risk of subsequent opioid misuse in a cohort followed to adulthood.(24) Even when used as prescribed, opioids arent harmless; side effects include nausea, constipation, lethargy, and respiratory depression. Most patients who chronically use opioids were originally prescribed the medication for acute pain. Until recently, it was believed that patients prescribed opioids for an acute problem were unlikely to develop drug abuse or addiction.(25,26) Current systematic reviews indicate that the evidence for that opinion would not meet current scientific standards. (27,28) Younger age, illicit drug use, tobacco use, alcohol abuse, and family history of drug and alcohol use are all risk factors for prescription drug abuse, along with social factors (unemployment, sexual abuse) and mental health conditions (e.g. depression, anxiety, and PTSD). (29,30) Due, in part, to these risks, the U.S Food and Drug Administration (FDA) recently (Feb 2016) announced an initiative to prioritize non-opioid alternatives for pain relief.(4)

Gaps in Evidence

Renal colic and Musculoskeletal Back Pain: Common Conditions with Variable Treatment Strategies

Acute kidney stone pain (renal or ureteral colic) and musculoskeletal back pain represent common reasons for patients to present to EDs and urgent care settings seeking pain relief. There are large variations and gaps in knowledge about the optimal medications for these conditions. A vital part of management for both conditions is rapid pain management, which can be achieved through the use of either non-steroidal anti-inflammatory medications (NSAIDS) or opioids. Although both classes of medication provide relief, there are tradeoffs for each. Renal Colic: Kidney stone pain, characterized by severe flank and back pain, affects 12% of the population worldwide and causes approximately two million outpatient visits per year in the United States.(31) A 2004 Cochrane review of 20 trials showed that both NSAIDS and opioids led to clinically important reductions in patient reported pain scores and a pooled analysis of six trials showed a greater reduction in pain for patients receiving NSAIDS compared to opioids.(32) A recent 2015 Cochrane review of 50 studies affirmed the efficacy of NSAIDS for acute renal colic.(33) Yet, there is still treatment variation around the optimal class of medications for this condition in acute care: a 2014 review of therapeutic approaches real colic in the ED found 12 articles examining the use of opioids in renal colic pain treatment and 24 articles on the use of NSAIDS, most published after 2004.(34) Clinical guidelines recommend the use of NSAIDS, but also identify opioids, as acceptable care for acute kidney stone pain.(35) Back pain: Patients with back pain present to EDs approximately 2.6 million times per year. (36) National treatment guidelines for patients with back pain in acute care settings urge providers to

determine if there is a non-opioid analgesic that would provide adequate pain relief, and to reserve opioid use for severe pain or pain that has not responded to other analgesics, and to prescribe the lowest dose of opioids at discharge. (10,37) There are known gaps in the dissemination, adoption, and implementation of these guidelines.(38,39) Based on the prevalence, variations in care, and severity of pain for these conditions, our study team, including patient investigators, selected acute renal colic pain and acute musculoskeletal back pain as the most appropriate model conditions to evaluate a risk communication approach to treating patients with pain in acute care settings.

Risk Communication for Pain Control in Acute Care:

Providers often make therapeutic decisions, particularly around analgesia, without engaging patients. Moreover, when they do discuss risks and benefits to specific options, the communication is frequently devoid of context and probabilistic in nature (presenting the likelihood of outcomes using either descriptive words or numbers).(40,41) A recent emergency department randomized controlled trial demonstrated that a fact-based, literacy-appropriate, information sheet did not improve patients knowledge and safe use of opioid analgesics compared to usual care.(42) Narrative communication can be an inexpensive, sustainable, alternative method to disseminate and promote engagement around health information and to enhance other forms of risk communication. Narratives are defined as coherent stories with identifiable beginnings, middles, and ends that provide information about scene, characters and conflict; raise unanswered questions or unresolved conflict; and provide resolution.(43) These types of stories have been noted to improve the communication of health information by holding peoples attention and transporting their mental state.(44,45) Narratives, when combined with probabilistic communication, are recognized as an effective tool to promote health behavior change.(46,48) The addition of probabilistic information to narratives also decreases the bias associated with narrative information alone.(49,50) However, the role of narratives for communicating and translating risk evidence, specifically when attempting to improve care in acute care settings or for pain treatment, has not been evaluated in a comparative manner.(43,45,47,51)

*See references for Background at bottom of document

CHARACTERISTICS OF THE STUDY POPULATION:

1. Target Population and Accrual:
The target population is female or male ED patients 18 years to less than 70 years old presenting in the ED with 1) acute renal colic or 2) musculoskeletal back pain. Patients must be alert and awake, not hemodynamically compromised or in severe distress, not pregnant. Patients must speak and read English, must be cognitively intact, and must have their pain controlled sufficiently to the extent that a planned discharge is deemed safe by the provider. We will not sample patients who are currently taking opioids for chronic pain (defined as greater than 3 months) or to treat their cancer, which will be confirmed through a chart review of the patients medication list. Patients must also have access to a computer or mobile device that will allow them to conduct the follow up surveys.

To determine if an adequate number of patients would meet eligibility criteria, we conducted a detailed exploration of ED medical record data in the last year. We found 5479 potentially eligible individual patients with musculoskeletal back pain or renal colic (determined by ICD-9 code) at the 3 sites; 92% had back pain and 8% had renal colic. Based on these estimates, we will be able to recruit from a population of approximately 11,000 eligible patients presenting during the planned 2 year data collection period. While opioids were used frequently while patients were in the ED, opioid prescribing patterns on discharge differed widely by hospital (overall 20%: range 15-35%) and condition (overall, 20% received an opioid on discharge: back pain 17%, kidney stones 52%). For further information on our planned enrollment in keeping with the principles of a PCT, the eligibility criteria will be broad. Acute care patients at one of 9 enrollment sites (3 hospitals), aged 18-70 with a diagnosis of acute renal colic or musculoskeletal back pain, will be eligible for enrollment. Patients must speak and read English, must be cognitively intact, and must have their pain controlled sufficiently to the extent that a planned discharge is deemed safe by the provider.

2. Key Inclusion Criteria:

- Age 18 years to 70 years old.
- Experiencing back pain or renal colic based on provider assessment
- Text messaging and internet access including email capabilities or access to a smartphone
- Anticipated discharge within 24 hours.

3. Key Exclusion Criteria:

- Patients who take opioids for chronic pain or cancer treatments.
- Patients who have taken opioids in the past month with the exception of patients who have taken opioids in the previous 48 hours before arriving at the ED for their presenting condition based on provider assessment.
- Patients who are pregnant, in police custody, intoxicated, cognitively impaired, or otherwise unable to fully consent and participate based on provider assessment
- Patients who are hemodynamically compromised, in respiratory distress, or in severe emotional or physical distress based on provider assessment.
- Patients older than 70 or younger than 18.
- Patients who will be admitted to hospital or deemed to have a critical illness based on provider assessment.
- Patients who are cognitively impaired based on provider assessment.
- Patients who are suicidal or have homicidal ideation by chart review and clinician assessment
- Patients with evidence of aberrant behavior based on provider assessment.
- Patients who do not have a phone, text messaging OR email address
- Patients under police arrest at ED visit
- Patients who are non-English
- Patients previously enrolled
- Patient with any current contraindications for NSAIDs or opioid medications including allergies, chronic kidney disease (GFR 60, if measured).
- Patients with a diagnosis of back fracture based on provider assessment.

4. Subject Recruitment and Screening:

In order to recruit participants for this study, a research assistant, research coordinator or an Academic Associate will review the electronic medical record (EMR) track board to identify patients who meet the above eligibility criteria in the Emergency Department 7A-12A Monday-Sunday. Research assistants will be trained on which components of the electronic medical record should be reviewed to determine eligibility (their chief complaint, age, and their medication list to confirm they are not on opioids on a chronic basis). Research assistants will then approach all potentially eligible patients in their room using the consent form and make sure that they do not have any exclusion criteria. If they are eligible and willing to participate, the RA will obtain written consent. Northwell policy requires participants receive a signed copy of the consent form. To meet this requirement, participants enrolled at the Northwell sites will fill out two copies of a paper consent form, which will be then signed by a witness and by the consenting investigator, and dated by each respective signee. One copy will be given to the participant and one copy will be maintained for study records. No personally identifying information will be collected before the consenting process. Once consent is signed, participant's first name, last name, and date of birth will be provided to U Penn to open an account on Way to Health Portal. Participants will then sign e-consent on the platform, consenting them to participate in the study. All participants providing consent will then complete a baseline survey and randomized to 1 of 3 trial arms. Patients not interested will be thanked for their time.

5. Early Withdrawal of Subjects:

Participants can withdraw from the study at any point from the study.

6. Vulnerable Populations:

Children, pregnant women, fetuses, neonates, or prisoners are not included in this research study.

7. Populations vulnerable to undue influence or coercion:

The eligible patients may be Penn employees or their family members. To decrease the risk of coercion or undue influence, the study member enrolling patients will specifically emphasize that participation is voluntary. The consent form also notes that participation is voluntary.

STUDY DESIGN:
We are enrolling patients experiencing acute pain whose primary complaint is renal colic or patients with musculoskeletal back pain in the Emergency Department ages 18 years to less than 70 years old. Once e-consented, a member of the research team will have each patient set up a profile on the iPad using Way to Health (WTH), a web-based platform to conduct the intervention. It is within this platform that the e-consent form with patients are reviewed. Patients who provide written informed consent using WTH will complete an informational survey that asks about demographics and they will be randomized to 3 trial arms for the Randomized Practical Clinical Trial (PCT). Randomization also takes place through WTH. Patients will then be followed up via a text message survey daily from days 1-7, at day 14, and then at 3 months, they may receive text message, email, and/or phone call reminders to complete a 3 month survey which they can choose to complete via text, email, or phone call. We also intend to follow up with patients at month 12 after enrollment. All of the surveys at baseline and follow up will be administered through WTH.

Incentives will be provided using a reloadable bank gift card upon completion study activities. This cash card will work through the Greenphire clincard system (SOP attached to this submission). Information about the Greenphire system will be provided in the consent document for the participant. Information will be collected from the participant in order to set up an online account that the participant will be able to access. At the Northwell Health site, participants will sign a written copy of the consent form prior to providing any information on the Way to Health platform.

With the exception of the electronic follow-up via text or email, all research activities will be conducted in the HUP Emergency Department and Emergency Department at Presbyterian Hospital, the Mayo Clinic Emergency Department, the North Shore University Hospital Emergency Department, the Long Island Jewish Medical Center Emergency Department, and the Emergency Department at the University of Alabama-Birmingham and will be conducted private settings. All research staff will be fully trained in the protocol and they will be supervised by the PI, Dr. Zachary Meisel, and the site PIs.

METHODS:

1. Study Instruments:

We will use a series of widely recognized, accepted, standard surveys that measure patient reported outcomes of opioid or other pain relief usage.

To measure days to no opioid use, we will ask the number of pain meds taken daily. This will be collected at days 1-2, 4-6, day 14, 3 months.
To measure self-reported opioid use, we ask the type and number of pain meds taken daily at days 1-2 and 4-6, day 14, 3 months.
To measure functional status, will use the Back Pain Functional Scale at, days 1, 7, 14 and at 3 months. (1)
To measure patient reported preference for pain relief, we will use the Patient pain relief preference survey at baseline.
To measure satisfaction with pain treatment, we will use the American Pain Society Patient Outcome Questionnaire at Baseline, Days 1 and 7, Day 14, 3 months (2).
To measure trust in providers, we will use the Trust in Physician Scale at day 7 (3).
To measure follow-up visits for pain, we will use the Self-Report additional provider visits at day 14 and 3 months.
To measure patient-reported measures of shared decision making, we will use CollaboRATE at day 1 (4).
To look at agreement on pain treatment between patient preference and provider decision, we will compare patient preference to the EMR documentation at baseline.
To measure the probability of becoming addicted to opioids, we will use the Opioid Risk Tool (ORT) at baseline (5).
We will also provide a visual tool that we have created to communicate risk of opioid addiction to Arms 2 and 3 based of their score of the ORT.

We will then use the Risk Assessment Recall, to see if patients recall their risk level for those who were given their scores at baseline. This will be used at Day 14 and 3 months.
We will also administer and Informational survey where we will collect demographic information at baseline (6)(7)(8).
We also use an additional survey, the SOAPP®-R only at Penn, to measure risk of addiction to opioids at baseline (9).
To measure health literacy, we will administer a short health literacy survey on Day 3(10).
To measure health numeracy, we will administer the Subjective Numeracy Scale on Day 3 (SNS) (11)
To measure health status, we will use a self-rated health survey, the MOS-20 at baseline and at 3 months (12).
To measure misuse of opioids, we will administer the Current Opioid Misuse Measure (COMM) at 3 months. (13).
To see how many and which videos participants watched, we administer a Video Survey to arm 3 participants on days 2, 4-6 and day 14.

*See references for Study Instruments at bottom of document

2. Group Modifications:
Since each participant will be receiving the same survey instruments, there will be no group modifications.

3. Method for Assigning Subjects to Groups:
All ED patients that potentially meet inclusion criteria will be tracked by an RA during data collection hours. A research assistant will systematically approach all patients who potentially meet inclusion criteria during specified data collection time frames tracking if they meet exclusion criteria and/or are willing to enroll in the PCT study. Study participants who provide written consent and complete the baseline survey will then be stratified by condition (back pain and kidney stone pain) and then randomized in blocks of 15 into 1 of 3 trial arms.

4. Administration of Surveys and/or Process:
All participants, regardless of trial arm, will complete an informational survey on day of enrollment and will participate in electronic follow-up via text message reminders and/or email messages daily for 7 days, then on day 14. At 3 months, they will receive a phone call to solicit their opinion of the text/email follow up process. Finally, we hope to meet or call participants at one year.

5. Data Management:
Non-electronic data will be entered using database forms created by the study team and database manager using REDCap (Research Electronic Data Capture) from the initial demographics collected at enrollment and the patient opioid risk assessment to the last telephone interaction. REDCap is a secure, web-based application designed to support data capture for research studies, providing 1) an intuitive interface for validated data entry; 2) audit trails for tracking data manipulation and export procedures; 3) automated export procedures for seamless data downloads to common statistical packages; and 4) procedures for importing data from external sources.106 REDCap exports directly into a variety of data management and analytic software, such as Excel, SAS, Stata, R, and SPSS. In REDCap, data fields can be pre-specified as patient identifiers, and data exports can be done with those fields excluded. Additionally, REDCap fields can be used to ensure data quality through validation tools, such as a numeric range or the type of value entered (i.e. date/time field), which can be limited prior to the start of data entry, and the research assistant will be prompted to fix the erroneous value in real-time before moving on. 106 . A 20% sample of data will be entered twice by separate data entry clerks for data verification. If there is more than a 5% data entry error, all forms will be double entered. All data files created and modified online are automatically backed up onto a secure server maintained by the REDCap Administrator at the University of Pennsylvania. All electronic follow-up information will be stored electronically and automatically filter into a Password-protected database.

All source documents will be identified by study identification (ID) number, and the key to that ID number will be kept in a locked file cabinet. All personally identifiable information also will be kept separately in a locked file cabinet. No results will be reported in a personally identifiable manner. Participants' survey information will be collected via WTH on a tablet computer. The tablet will be password protected and data will be safely transferred to a protected server daily. All tracking system data and research database information will be password-protected with several levels of protection: first, a password will be required to access the computer of the user who has access to the database; second, a password will be required to access the database. Participants who

agree to the electronic follow up will be prompted by text message (or email if preferred) to respond to follow-up electronic questions daily for 7 days after their ED visit, 14 days after visit and 3 months after their visit, they will receive a phone call. The possible questions and various combinations of questions that we will ask through text message are attached (See daily text questions attached).

Contingent upon final approval from the IRB, the study team will make data available to other research entities as the PI deems appropriate. This includes internal data sharing at the University of Pennsylvania and other sites, as well as external data sharing such as with academic research journals that request data prior to publishing manuscripts. For open source publishers, only de-identified data cleaned of dates and geographic information greater than area code will be shared.

6. **If Applicable (if not delete the section): Management of Information for Multi-Site Research where a Penn Investigator is the Lead Investigator of a Multi-Site Study, or Penn is the Lead Site or Coordinating Center in a Multi-Site Study.

Three additional sites will be enrolling patients as part of this protocol: Mayo Clinic located in Rochester,Minnesota, the University of Alabama-Birmingham, and North Shore University Hospital and Long Island Jewish Medical Center in Manhasset, New York. The protocols at the other sites will be coordinated in advance of patient enrollment at all sites. All study documents will be finalized at Penn and approved by the Penn IRB (when required) prior to distribution to other study sites for local submission. Penn-approved consent documents will be sent to sites to use as a template in developing their own forms. Tracking spreadsheets will be developed to track the local IRB approval for all documents at each site. This will apply for initial submissions, as well as any modifications.. Each site will also be conducting enrollment at their ED settings. The trial PI is responsible for reporting unanticipated problems, reporting interim results, and coordination of protocol modifications. The University of Penn will be responsible for conducting all data management and analyses.

7. Subject Follow-up:

All participants who provide written consent and are randomized, regardless of trial arm, will participate in electronic follow-up via text message reminders for the first 7 days after enrollment, then again on day 14. At 3 months after enrollment, participants will receive a follow-up survey via text message, email and/or phone call. We will also follow-up at 12 months. Research Assistants will ask patients to provide their cell phone number or email address to receive text messages after discharge from the ED.

STUDY PROCEDURES:

1. Detailed Description:

Research participants will be approached in their room at the ED for enrollment. If they provide written consent, they will be randomized into 1 of 3 trials arms (Figure 1: Study Enrollment Protocol).
Arm 1: Generalized Risk Communication (GRC): Participants in this arm will receive standard discharge instructions similar to instructions they would receive during usual care. This arm represents a standardized way of communicating post-discharge risk-benefit information about treatment options for patients with back pain and renal colic. The GRC, includes a standardized discharge information sheet about the clinical condition of interest and a written overview of population based evidence describing comparative benefits and side effects of alternative classes of medication acute pain (GRC attached to this protocol).

Arm 2 Probabilistic Risk Communication (PRT): The probabilistic risk communication tool (PRT) is a visual tool that communicates risk using the previously validated Opioid Risk Tool (ORT). The ORT is designed to assess risk of opioid dependency for patients for whom an opioid pain relief prescription is being considered in outpatient settings. Patients in this arm will be given an iPad which will prompt them to take a short survey that automatically communicates their risk score. After which the iPad will show them a color coded visual thermometer that informs them of their risk of having issues related to opioids (PRT image attached to the protocol).

Arm 3: Narrative Enhanced Risk Tool (NERT): Participants assigned to this arm will receive the PRT described above but will also be instructed to watch one or more narrative videos. This video intervention will include a brief narrative video of a patients experiences of pain and pain treatment. Narrative videos are developed from actual stories - put into a in a structured format of ~ 2-minute length and recorded using actors. The video will also mention the risks of keeping unused opioids at home, including the risk of diversion.

After enrollment, patients will be followed up with electronically via text message reminders for the first 7 days after enrollment, then again on day 14 and 3 months after enrollment. If patients do not respond to the initial text message to complete the surveys, they may receive an additional text message, email or phone as a reminder to complete the surveys.

2. Data Collection:

All personal information that the participant is asked to provide will be collected via *Way To Health. Way To Health* collects subjects' names, dates of birth, addresses, email addresses, and phone numbers.. They also request the name and phone number of an alternate contact. To assure that participant confidentiality is preserved, individual identifiers are stored in a single password protected system that is accessible only to study research, analysis and IT staff. An investigator or statistician who logs in will be able to access only non-identifiable data. The *Way To Health* administrative group and research coordinators responsible for contacting participants for follow-up study visits or responding to questions about the study are able to view participant names and contact information. The WTH web development team and Project Director currently have administrative access to PHI. All of these personnel will have completed Human Subjects Protection and HIPAA privacy training. The system automatically generates logs of all data queries which can be reviewed by research staff to ensure that no unauthorized persons have gained access to identifiable information. This system is hosted on site at The University of Pennsylvania (UPenn) and is protected by a secure firewall and several layers of operational security. Once a participant has been entered into this system, they are given a unique study identification number (ID). Any datasets and computer files that leave the firewall are stripped of all identifiers and individuals are referred to by their study ID. The study ID is also used on all analytical files.

The Penn Medicine Academic Computing Services (PMACS) is the hub for the hardware and database infrastructure that supports the project and the *Way To Health* web portal is built on this infrastructure. The data collected for *Way To Health* based studies is stored in mySQL databases on a PMACS-operated blade server environment devoted specifically to *Way To Health*. The data center is housed in Information Systems and Computing at 3401 Walnut Street. All data are stored in a single relational database, allowing researchers to correct mistakes. Every SQL transaction, including accessing and changing data, is logged for auditing purposes. Data are entered into the database through several different mechanisms. Participants enter their own personal information and respond to surveys through a PHP-based web interface. Researchers have a separate interface that allows them to manually enter data if needed. Datasets are stripped of all personally identifiable information when exported for analysis. The web application automatically removes all identifiers when a researcher requests an analytic dataset. The only people with access to identifiable participant information are pre-specified Research Coordinators responsible for contacting participants for follow-up. Personal information and research data will be stored in separate SQL tables and will be linked by a computer-generated ID number. Additionally, any information that leaves this system to communicate with third party data sources (i.e. survey software) is stripped of any identifiers and transmitted in encrypted format. The same unique study ID is used to link these outside data to the participants. All data for this project will be stored on the secure/firewalled servers of the PMACS Data Center, in data files that will be protected by multiple password layers. These data servers are maintained in a guarded facility behind several locked doors, with very limited physical access rights. They are also cyber-protected by extensive firewalls and multiple layers of communication encryption. Electronic access rights are carefully controlled by UPenn system managers. *Way To Health* uses highly secure methods of data encryption for all transactions involving participant's financial information using a level of security comparable to what is used in commercial financial transactions. This multi-layer system of data security, identical to the system protecting the University of Pennsylvania Health Systems medical records, greatly minimizes the risk of loss of privacy. All communications between users and our site will be encrypted with SSL/HTTPs technology.

3. Genetic Testing:

Not applicable

4. Use of Deception:
This study does not include use of deception.

5. Statistical Analysis:
Standard statistical tests (2 and ANOVA or Kruskal Wallis test) will be performed to determine outcome distributions and whether patients differ with regard to demographics in the 3 study arms. To test the hypothesis that patients in the NERT group will take significantly less morphine equivalents than patients in PRT group, the primary analytic technique will be a Poisson (log-linear) regression or a Negative Binomial (ZINB) regression.101 This technique was chosen since count data is not normally distributed and with our primary outcome may be zero-inflated. The outcome distribution will determine the best model to be used: Poisson if conditional variance does not exceed the mean or ZINB for over dispersed data (variance much large than mean). Models will be developed using SAS procedures GENMOD or COUNTREG using a log link and Poisson or a ZINB distribution. All models will be adjusted for baseline pain level, condition, and any complications, as well as possible modifiers/confounders such as age, gender, race, education, employment, insurance, income (SES), and marital/relationship status. Additionally we will assess differences in number of days to no/minimal pain (score of 0/1 out of 10 on adequacy of pain management) and number of days to cessation of opioid use using Kaplan-Meier curves and Cox proportional hazard regression. Kaplan-Meier curves were chosen instead of ANOVA in repeated measures as it is possible that not all patients will have achieved minimal/no pain and/or be off opioids and therefore data will be right censored. This type of analysis also accommodates patients that are lost to follow-up more easily than an ANOVA as they will be censored at the time they were last known to respond to the electronic log. The log rank test will be used to assess differences in rate of cessation of opioid use between the 2 groups. To assess short term changes (7 days and 2 weeks) in adequacy of pain management and cessation of opioid use between the 2 groups, we will use mixed effects models, as we are interested in the rate to pain relief and/or to cessation, regardless of whether total pain/relief or cessation of opioid use was achieved during these shorter time periods. To test the hypothesis that patients in the NERT group will return to usual activities sooner than those in the PRT or GRC groups, Kaplan-Meier curves and the Cox proportional hazards models will be used to assess difference in time to return to functional status over the 3 month study period. Functionality outcomes will be defined as time from the ED visit to the first electronic log that a patient reports a 0/1 (corresponding to how much pain interferes with usual activities none/minimal) on the functionality scale. The log rank test will be used to assess differences in rate of return to functionality between groups. Since it is expected that condition (back pain vs renal colic) will differ in their rate of return to usual activities, analyses will be stratified on condition. To assess short term changes (7 days and 2 weeks) in functionality in the 2 groups, we will again use mixed effect models. For these analyses, we are interested in the rate of change, regardless of whether full functionality is achieved. To accommodate more than one predictor variable, time to functionality will be assessed using Cox Proportional Hazards regression. Using the bivariate KaplanMeier curves as a starting point, significant predictor variables will be entered into a proportional hazards model and tested for association with time to functionality. All models will be forced entry with backward elimination. Additionally, at the 3 month interview, functionality will be assessed with BPFS and MOS-36 scales90,104. For this analysis, a 2 way ANOVA (group and condition as main effects) will be used. Similarly, trust in the provider using the trust in physician scale (range of scores 11-55),and patient shared decision making using the CollaboRATE scale, the 2-way ANOVA will be performed at 3 months and at discharge, respectively. To test the hypothesis that patients in the video narrative group will have greater satisfaction with adequacy of pain management, 2 analytic techniques will be used. Satisfaction will be assessed using selected items from the American Pain Society Patient Outcome Questionnaire. To assess differences in the absolute score at 2 weeks and 3 months between the 2 groups, an analysis of covariance will be performed with group and condition as main effects, an interaction term crossing approach with condition, and covaried on baseline scores. In addition to this analysis, patient satisfaction will be dichotomized; 1-3 (not satisfied), 4-6 (satisfied) and a generalized linear model (SAS GLM procedure) with a log link, Gaussian or binomial error, and robust estimates of the standard errors of the model coefficients102 will be employed. This technique will allow for the calculation of relative risks (RRs) as opposed to odds ratios in logistic regression which tend overestimate relative risk when the outcome of interest is common. It will also give us the flexibility to compare the 2 groups within one model while adjusting for other possible predictor variables. Entrance of covariates into the model will be determined by assessing the association of these covariates with the primary outcome using bivariate analyses, such as chi-square and Fishers exact tests (2X2 tables). Finally, to

assess agreement between Patient Preference and Provider Decision, for the 4 possible options (eliminates let my provider decide or no choice), a weighted Cohens kappa will be calculated for each treatment arm.

RISK/BENEFIT ASSESSMENT:

1. Risks:
Due to the nature of the population, patients may be experiencing pain when approached for enrollment, or alternatively may have received sedative medications in the ED. To address this we will not approach patients until they have been clinically assessed and treated for pain. Patients will only be approached once alert, awake, oriented, able to consent and safe for discharge (provider determined). Subjects are at risk for emotional distress from disclosing personal information asked in the baseline and follow-up interviews. While we do not specifically ask, any patient who spontaneously self-identifies as suicidal, homicidal or a child abuser would be at risk of involuntary confinement, involvement of child protective services, or other protective action. There is also the outside risk of re-identification (breach of confidentiality) of research data, as this risk must be considered for any study using data that is stored on a computer.

2. Benefits:
A fundamental conflict in pain management between the need for opioid pain medication as part of compassionate care and concern for the public health crisis around opioid misuse, drug diversion, overdoses, and deaths exists. There are no tested strategies for relieving acute pain that also addresses the prevention of misuse of opioids in high-risk patient populations. The objective of the proposal is to identify whether the video narrative intervention in conjunction with the probalistic risk communication tool is more effective than probabilistic risk communication alone or a generalized risk communication information sheet for patient centered outcomes. The proposal builds on the premise that patients with greater awareness of how opioids work and the risks involved in taking them will use them more judiciously and have improved functional outcomes. Potential benefits include improved knowledge, risk awareness, and reduced use of addictive medication. The results have the potential to improve care for patients with acute pain and to broadly change clinical practice to reduce opioid misuse and death. Moreover, participation is contributing to the overall knowledge of prevention interventions which may have benefits to society and public health in general.

3. Subject Privacy:

All participation is voluntary, and in-person surveys will be conducted in a private space at the Emergency Department. The full consent process will take place. For Northwell, no identifying information will be collected before the complete consenting process. If participants no longer wish to participate, the study team will no longer contact them and text-message follow up will cease immediately. Participants are reminded at the end of each text message survey that they may opt out by contacting the study team.

4. Subject Confidentiality:

How will confidentiality of data be maintained? Check all that apply.

☒ Paper-based records will be kept in a secure location and only be accessible to personnel involved in the study.

☒ Computer-based files will only be made available to personnel involved in the study through the use of access privileges and passwords.

☒ Prior to access to any study-related information, personnel will be required to sign statements agreeing to protect the security and confidentiality of identifiable information.

☒ Whenever feasible, identifiers will be removed from study-related information.

☒ A Certificate of Confidentiality will be obtained, because the research could place the subject at risk of criminal or civil liability or cause damage to the subject’s financial standing, employability, or liability.

☐ A waiver of documentation of consent is being requested, because the only link between the subject and the study would be the consent document and the primary risk is a breach of confidentiality. (This is not an option for FDA-regulated research.)

☒ Precautions are in place to ensure the data is secure by using passwords and encryption, because the research involves web-based surveys.

☐ Audio and/or video recordings will be transcribed and then destroyed to eliminate audible identification of subjects.

☐ Other (specify):

The study involves data collected with protection of confidentiality. All questionnaires will be filled out in private. The data management team will remove any identifying information when information is entered into a database. Study and survey data collected during the baseline and subsequent visits will be kept physically and electronically secure. All data will be stored on an institutionally managed device and server. Completed questionnaires will be kept in a locked and secured area in the locked research study office. Subject names will not appear on these forms. Likewise, any data entered into a database will not have the patients name on it. The key to link the subject ID number to the rest of the study information will be kept by the researchers in a separate locked file cabinet, along with consent forms and contact information for follow up. This information will be kept locked separate from the rest of the subject information and interview data and identified only by the anonymous subject ID numbers. The screening/contact forms will be kept until the follow-up period is complete, after which time the name portions will be removed and destroyed. No one but the PI, project manager, and study staff responsible for the follow up will have access to these files. Any data used for results will only be presented in a de-identified manner in the aggregate.

5. Protected Health Information

We will collect the following information:

- Name
- Street address, city, county, precinct, zip code, and equivalent geocodes
- All elements of dates (except year) for dates directly related to an individual and all ages over 89
- Telephone numbers
- Electronic mail addresses
- Medical record numbers
- Health plan ID numbers

6. Compensation:

Once consented and enrolled, all subjects will participate in a baseline assessment; and enroll in an electronic follow-up system (text/email and electronic daily log via calendar app/web interface or calendar app), and be registered for a Greenphire ClinCard for wireless compensation for completed study activities.

Participants will be paid $5 at enrollment. Participants will then receive $2 for 7 daily text messages plus an additional $5 ($19 total) if they complete each test message. They will also receive $20 to complete the 2-week (day 14) text message, and $40 complete the 3-month survey. In total participants may receive up to $84 dollars for completion of all study activities.

7. Data and Safety Monitoring:

The study involves data collected with protection of confidentiality. All questionnaires will be filled out in private.

Study and survey data collected during the baseline and subsequent visits will be kept physically and electronically secure. Completed questionnaires will be kept in a locked and secured area in the locked research study office. Subject names will not appear on these forms. Likewise, any data entered into a database will not have the patient's name on it. The key to link the subject ID number to the rest of the study information will be kept by the researchers in a separate locked file cabinet, along with consent forms and contact information for follow up. This information will be kept locked separate from the rest of the subject information and interview data and identified only by the anonymous subject ID numbers. The screening/contact forms will be kept until the follow-up period is complete, after which time the name portions will be removed and destroyed. No one but the PI, project manager, and study staff responsible for the follow-up will have access to these files. Any data used for results will only be presented in a de-identified manner in the aggregate.

8. Investigator's Risk/Benefit Assessment:

In general, the benefits outweigh the risks in this study. Participants will receive information on their risk of opioid misuse while achieving adequate pain relief. Participants are also contributing to preparation of a larger study, which in turn may have benefits to society in general.

Recruitment and Informed Consent: Patients will consent via e-consent on the Way to Health Platform.The e-consent form outlines all of the above risks.Trained staff, supervised by experienced clinicians, will inform participants regarding the purpose of the study. The staff will emphasize that participation is voluntary, the participant may stop at any time if he or she chooses, and that care will in no way be adversely affected by their choices related to study enrollment. Research assistants will be trained to approach and describe the study in a uniform manner, taking care to not present one type of pain management over another as more beneficial or less risky. As with all research conducted at UPHS, compassionate medical care takes precedence over all study involvement. Emergency Department providers are trained to follow the Standard Opioid Prescribing Protocol, which provides clear discharge instructions about appropriate use of opioids and non-opioid pain medication and risks of opioid dependence, addiction, diversion, and overdose.
At the Northwell site specifically, participants will consent both via e-consent on the Way to Health Platform, and as per Northwell's HRPP's guidelines, participants enrolled at the Northwell sites will also receive a signed copy of the consent form. One copy will be given to the participant and one copy will be maintained for study records. This process will occur prior to collecting any information on the Way to Health platform. Both the e-consent form and the written consent forms outline all of the above risks
Protection Against Suicidal/Homicidal Risk: Subjects will be excluded from the study and will be appropriately linked to needed psychiatric or other services if they are clinically judged (by the treating provider) to be homicidal, suicidal, actively psychotic, or otherwise unable to give informed consent due to a cognitive or mental health issue. Research assistants will be trained to assess for exclusion criteria and they will always have access to on-site clinical supervisors. Any subject with homicidal or suicidal risk will be evaluated immediately for appropriate psychiatric care by the clinical care team. Drs. Meisel, Perrone, Becker, and Hess are familiar with and competent in the process of psychiatric assessment and involuntary commitment, in the unlikely situation that an enrolled individual needs this psychiatric care. As PI and an ED physician, Dr. Meisel will take primary responsibility for all safety issues related to the study. All sites have psychiatric services readily available should it be necessary to engage their assistance. Any patient who becomes emotionally distressed during the study will be referred immediately to a Penn psychiatrist, who will provide additional consultation, supervision and, if needed, medication. The research team will review the cases of any enrolled subjects who are referred or considered for potential psychiatric admission. Any potential serious adverse events related to being a subject in the research study will be reported within 24 hours to the IRB.
Protection Against Risks During Follow Period: Risks for participating in the study are minimal and are unlikely to increase during follow-up. If patients have concerns about opioid dependency, they will be given a primary care referral and resource lists for any needed community-based social service resources, substance abuse treatment, and medical resources. No patients will be encouraged or discouraged from taking more or less of a particular pain medication by the study team. RAs collecting the baseline and follow up data will specifically be trained to be neutral on type and amount of medication taken. Written discharge instructions given to all patients at discharge inform them about the range of medication and complementary non-medication options for pain management. We anticipate the patient playing a larger role in their pain management plan will allow for more open conversations with their providers about medications. All participants will be given study contact information if they need additional assistance.

Certificate of Confidentiality: Dr. Meisel will obtain a Federal Certificate of Confidentiality prior to the study start. We recruit in private and do not disclose to anyone outside the research team the identities of participants. Limitations to Confidentiality: All enrolled patients will be informed verbally and as part of the written consent that they are free to skip any survey questions or to withdraw from the study at any point. They will be informed that the information they provide will be held in confidence to the extent that the law allows under the Certificate of Confidentiality, but that the exception to this confidentiality is any disclosure of potential for immediate harm of themselves or others. Subjects will be notified prior to participation that if any of these issues are raised, the researchers will take whatever steps are necessary to protect the subject or others, including bringing the risk of harm to the attention of the proper authorities.

Training and Supervision of Research Assistants: All RAs complete IRB certified training regarding human subjects protection and are thoroughly trained and supervised in the conduct of the research protocol. A project manager, responsible for general oversight of the project as a whole, will ensure all study procedures are followed, and any issues are brought to the attention of the Investigators and resolved in a timely manner. RAs working on this proposal will be trained to demonstrate sensitivity to pain experience of patients and to neutrally present the intervention tools. Scripts and role playing will be used when training research assistants how to approach patients, when presenting the study, and on maintaining a nonjudgmental neutral approach to collecting information on amount and type of pain medication taken, history of substance abuse, and other sensitive psychosocial risks.

Assurances**:** The University of Pennsylvania has agreed to use Federal Certificates of Confidentiality to protect against the compelled disclosure of personally identifiable information and to support and defend the authority of the Certificate against legal challenges. All subjects will be informed that a Certificate has been issued, and they will be given a description of the protection provided by the Certificate.

Potential Benefits: The objective of the proposal is to identify whether the video narrative intervention in conjunction with the risk communication tool is more effective than risk communication alone or generalized risk communication in patient outcomes. Potential benefits include improved knowledge, risk awareness, and reduced use of addictive medication. Moreover, participation is contributing to the overall knowledge of prevention interventions which may have benefits to society and public health in general.

Importance of the Knowledge to be Gained: A fundamental conflict in pain management between the need for opioid pain medication as part of compassionate care and concern for the public health crisis around opioid misuse, drug diversion, overdoses, and deaths exists. There are no tested strategies for relieving acute pain that also addresses the prevention of misuse of opioids in high-risk patient populations. The proposal builds on the premise that patients with greater awareness of how opioids work and the risks involved in taking them will use them more judiciously and have improved functional outcomes. This research will empirically evaluate two innovative low cost interventions with ED patients presenting with acute pain. The results have the potential to improve care for patients with acute pain and to broadly change clinical practice to reduce opioid misuse and death.

INFORMED CONSENT:

1. ***Consent Process:***

Patients who are interested in participating will be asked to enroll in Way to Health. Once they create a profile in ay to Health, patients will be read the consent form via iPad and will sign the consent form electronically on the iPad. . Patients will then be asked to complete an informational survey and be randomized into 1 of 3 trial arms. Patients will be informed that they may refuse to answer any question for any reason, can skip any question and can stop at any time. We will tell participants that we are not using the data for diagnostic purposes. By signing the written consent forms, patients give permission for follow-up and access to their Electronic Medical. ED RAs will be responsible for reviewing the electronic medical record (EMR) track board for patients recruited through the ED. See attached for written informed consent. At Northwell's site, participants enrolled at the Northwell sites will also fill out two copies of a paper consent form, which will be then signed by a witness and by the consenting investigator, and dated by each respective signee. One copy will be given to the participant and one copy will be maintained for study records. Both the e-consent form and the written consent forms outline all of the above risks. No identifying information will be collected before the participant has fully e-consented and signed both copies of the paper consent form, using best clinical practice. *All identifying contact information will be destroyed after all the follow up surveys have been completed.*

2. Waiver of Informed Consent:

No waiver of informed consent will be requested.

RESOURCES NECESSARY FOR HUMAN RESEARCH PROTECTION:

The research team brings together a strong group of clinician and patient researchers with a unique set of complementary skills, knowledge, analytic abilities and personal experiences that are ideal for testing the aims and disseminating the results of the proposed project (See Investigator Biosketches). Previous collaborative work by the investigators supports both the need for and their ability to carry out the proposed study. Zachary Meisel, MD, MPH, MS (PI) is an emergency physician and an expert on use of narratives for translating evidence-based interventions for the public. Erik Hess, MD in an emergency physician and clinical epidemiologist, and an expert on patient centered decision science in acute care. He serves as the PI of 2 PCORI-funded emergency department decision-aid based studies, one of which The Chest Pain Choice trial, has been successfully conducted at both the Mayo and Penn study sites. Dr. Hess is now the site PI at the University of Alabama-Birmingham. Venkatesh Bellamkonda, MD has taken over the role of site PI at Mayo Clinic. Dr. Bellamkonda is an emergency physician. Karin Rhodes, MD, MS has conducted 3 successful NIH funded RCTs involving 4 different EDs using patient-centered health information technology and identified key deficiencies in the quality of ED discharge instructions, including instructions about medication use. Lance Becker, MD temporarily took over as Site PI for Dr. Rhodes, while she completes a 1- year fellowship in Washington DC from September 2017-September 2018. Dr. Becker has been the PI more than 20 other research studies, including many conducted in ED settings, additionally, he is the Chairman of both ED recruitment sites at Northwell. As of July 1st 2019, Dr. Lance Becker has once again resumed his role as PI at Northwell Health because Dr. Karin Rhodes took another position. Jeanmarie Perrone MD, is a medical toxicologist, chair of the medical therapeutics committee, member of the NIDA-sponsored Penn Center of Excellence in Pain Education, and sits on the Drug Safety and Risk Management Advisory Committee for the FDA. Frances Shofer, PhD is an epidemiologist with expertise working with ED electronic medical record data and conducting ED studies, including a recent prescription opioid study. Marilyn M. Schapira, MD, MPH is an expert in health numeracy, risk communication, and the development and evaluation of decision support tools Carolyn Cannuscio, PhD. is an expert in qualitative methodology and narrative analysis with a strong interest in health disparities and community health. Erica Goldberg, MSW is the project manager for the study and an expert in qualitative methodology with a significant background in managing health service studies and quality improvement projects. Patient investigators: Jeff Bell, RN; Melissa Rodgers BA; Sharon McCollum, and Michael Zyla, BS, all have extensive experience as patients presenting with both acute and chronic pain and a variety of experiences with opioids, both good and bad. In addition, Rachel Graves, BA has personal experiences as a family member of a patient who struggled with opiate addiction. Together the team has created a balanced and evidence-driven approach to developing, refining, and testing optimal patient-centered risk communication tools.

References for Background

2. Paulozzi L, Baldwin G, Franklin G, et al. CDC Grand Rounds: Prescription Drug Overdoses - a U.S. Epidemic. MMWR Morb Mortal Wkly Rep. 2012;61(1):10-13. doi:mm6101a3 [pii].
3. Paulozzi L, Dellinger A, Degutis L. Lessons from the past. Inj Prev. 2012;18(1):70. doi:10.1136/injuryprev-2011-040294.
4. Califf RM, Woodcock J, Ostroff S. A Proactive Response to Prescription Opioid Abuse. N Engl J Med. 2016. doi:10.1056/NEJMsr1601307.
5. Substance Abuse and Mental Health Services Administration. The DAWN Report: Highlights of the 2011 Drug Abuse Warning Network (DAWN) Findings on Drug-Related Emergency Department Visit. Rockville, MD; 2013.
6. Levi, Jeffrey. Segal, Laura M. Miller A. Prescription Drug Abuse: Strategies to Stop the Epidemic 2013. Prescr Drug Abus Inj Policy Rep Ser. 2013:1-64. www.healthyamericans.org.
7. Holman JE, Stoddard GJ, Higgins TF. Rates of prescription opiate use before and after injury in patients with orthopaedic trauma and the risk factors for prolonged opiate use. J Bone Joint Surg Am. 2013;95:1075-1080. doi:10.2106/JBJS.L.00619.

8. Manchikanti L, Fellows B, Ailinani H, Pampati V. Therapeutic use, abuse, and nonmedical use of opioids: A ten-year perspective. Pain Physician. 2010;13(5):401-435. http://ovidsp.ovid.com/ovidweb.cgi?T=JS&CSC=Y&NEWS=N&PAGE=fulltext&D=emed9&AN=2010576242;\nhttp://gw2jh3xr2c.search.serialssolutions.com?url_ver=Z39.88-2004&rft_val_fmt=info:ofi/fmt:kev:mtx:journal&rfr_id=info:sid/Ovid:emed9&rft.genre=article&rft_id=info:
9. Volkow ND, McLellan T a, Cotto JH, Karithanom M, Weiss SRB. Characteristics of opioid prescriptions in 2009. JAMA. 2011;305(13):1299-1301. doi:10.1001/jama.2011.401.
10. Cantrill S V., Brown MD, Carlisle RJ, et al. Clinical policy: Critical issues in the prescribing of opioids for adult patients in the emergency department. Ann Emerg Med. 2012;60:499-525. doi:10.1016/j.annemergmed.2012.06.013.
11. Levi, Jeffrey. Segal, Laura M. Miller A. Prescription Drug Abuse: Strategies to Stop the Epidemic 2013. Prescr Drug Abus Inj Policy Rep Ser. 2013:1-64.
12. Weiss RD, Potter JS, Fiellin DA, et al. Adjunctive counseling during brief and extended buprenorphine-naloxone treatment for prescription opioid dependence: a 2-phase randomized controlled trial. Arch Gen Psychiatry. 2011;68(12):1238-1246. doi:10.1001/archgenpsychiatry.2011.121.
13. Motov SM, Khan ANGA. Problems and barriers of pain management in the emergency department: Are we ever going to get better? J Pain Res. 2009;2:5-11.
14. Wilson JE, Pendleton JM. Oligoanalgesia in the emergency department. Am J Emerg Med. 1989;7(6):620-623. doi:10.1016/0735-6757(89)90286-6.
15. Eder SC, Sloan EP, Todd K. Documentation of ED patient pain by nurses and physicians. Am J Emerg Med. 2003;21(4):253-257. doi:10.1016/S0735-6757(03)00041-X.
16. Fosnocht DE, Heaps ND, Swanson ER. Patient expectations for pain relief in the ED. Am J Emerg Med. 2004;22(4):286-288. doi:10.1016/j.ajem.2004.04.011.
17. Blank FS, Mader TJ, Wolfe J, Keyes M, Kirschner R, Provost D. Adequacy of pain assessment and pain relief and correlation of patient satisfaction in 68 ED fast-track patients. J Emerg Nurs. 2001;27(4):327-334. doi:10.1067/men.2001.116648.
18. Levi-Minzi MA, Surratt HL, Kurtz SP, Buttram ME. Under treatment of pain: A prescription for opioid misuse among the elderly? Pain Med (United States). 2013;14:1719-1729. doi:10.1111/pme.12189.
19. Bruckenthal P, Reid MC, Reisner L. Special issues in the management of chronic pain in older adults. Pain Med. 2009;10 Suppl 2:S67-S78. doi:10.1111/j.1526-4637.2009.00667.x.
20. Bekanich SJ, Wanner N, Junkins S, et al. A multifaceted initiative to improve clinician awareness of pain management disparities. Am J Med Qual. 2014;29(5):388-396. doi:10.1177/1062860613503897.
21. Gagnon AM, Kahan M, Srivastava A. Opioid use and abuse: is there a problem? Clin J Pain. 2007;23(8):661-662. http://www.ncbi.nlm.nih.gov/pubmed/17885343. Accessed February 9, 2016.
22. Bekanich SJ, Wanner N, Junkins S, et al. A multifaceted initiative to improve clinician awareness of pain management disparities. Am J Med Qual. 2014;29(5):388-396. doi:10.1177/1062860613503897.
23. Asim A, Gomes T, Zheng H, Mamdani MM., Juurlink DN., Bell CM. Long-term Analgesic Use After Low-Risk Surgery; A Retrospective Cohort Study. Arch Intern Med. 2012;172(5):425-430. doi:10.1001/archinternmed.2011.1827.
24. Miech R, Johnston L, Malley PMO, Keyes KM, Heard K. Prescription Opioids in Adolescence and Future Opioid Misuse. 2015;136(5). doi:10.1542/peds.2015-1364.
25. Portenoy RK, Foley KM. Chronic use of opioid analgesics in non-malignant pain: Report of 38 cases. Pain. 1986;25(2):171-186. doi:10.1016/0304-3959(86)90091-6.
26. J P, H J. Addiction Rare in Patients Treated with Narcotics. NEJM. 1980;302(2):123. doi:10.1056/NEJM198001103020221.
27. Ballantyne JC. Safe and effective when used as directed: the case of chronic use of opioid analgesics. J Med Toxicol. 2012;8(4):417-423. http://www.pubmedcentral.nih.gov/articlerender.fcgi?artid=3550253&tool=pmcentrez&rendertype=abstract. Accessed February 9, 2016.

28. Harden. Chronic opioid therapy: another reappraisal. APS Bull. 2002;12(1):8.
29. Edlund MJ, Martin BC, Devries A, Fan M-Y, Braden JB, Sullivan MD. Trends in use of opioids for chronic noncancer pain among individuals with mental health and substance use disorders: the TROUP study. Clin J Pain. 2010;26:1-8. doi:10.1097/AJP.0b013e3181b99f35.
30. Turk DC, Swanson KS, Gatchel RJ. Predicting opioid misuse by chronic pain patients: a systematic review and literature synthesis. Clin J Pain. 24(6):497-508. doi:10.1097/AJP.0b013e31816b1070.
31. Bensalah K, Tuncel A, Gupta A, Raman JD, Pearle MS, Lotan Y. Determinants of quality of life for patients with kidney stones. J Urol. 2008;179(June):2238-2243; discussion 2243. doi:10.1016/j.juro.2008.01.116.
32. Holdgate A, Pollock T. Systematic review of the relative efficacy of non-steroidal anti-inflammatory drugs and opioids in the treatment of acute renal colic. BMJ. 2004;328:1401. doi:10.1136/bmj.38119.581991.55 [doi]\rbmj.38119.581991.55 [pii].
33. Afshar K, Jafari S, Marks AJ, Eftekhari A, MacNeily AE. Nonsteroidal anti-inflammatory drugs (NSAIDs) and non-opioids for acute renal colic. Cochrane database Syst Rev. 2015;6:CD006027. doi:10.1002/14651858.CD006027.pub2.
34. Golzari SE, Soleimanpour H, Rahmani F, et al. Therapeutic Approaches for Renal Colic in the Emergency Department: A Review Article. Anesthesiol pain Med. 2014;4(1):e16222. doi:10.5812/aapm.16222.
35. Portis AJ, Sundaram CP. Diagnosis and initial management of kidney stones. Am Fam Physician. 2001;63(7):1329-1338.
36. Friedman BW, Chilstrom M, Bijur PE, Gallagher EJ. Diagnostic testing and treatment of low back pain in United States emergency departments: a national perspective. Spine (Phila Pa 1976). 2010;35:E1406-E1411. doi:10.1097/BRS.0b013e3181d952a5.
37. Chou R. Diagnosis and Treatment of Low Back Pain: A Joint Clinical Practice Guideline from the American College of Physicians and the American Pain Society. Ann Intern Med. 2007;147(7):478. doi:10.7326/0003-4819-147-7-200710020-00006.
38. Kilaru AS, Perrone J, Auriemma CL, Shofer FS, Barg FK, Meisel ZF. Evidence-based narratives to improve recall of opioid prescribing guidelines: A randomized experiment. In: Academic Emergency Medicine.Vol 21.; 2014:244-249. doi:10.1111/acem.12326.
39. Kilaru AS, Gadsden SM, Perrone J, Paciotti B, Barg FK, Meisel ZF. How Do Physicians Adopt and Apply Opioid Prescription Guidelines in the Emergency Department? A Qualitative Study. Ann Emerg Med. 2014. doi:S0196-0644(14)00221-2 [pii].
40. McCarthy DM, Cameron KA, Courtney DM, Adams JG, Engel KG. Communication about opioid versus nonopioid analgesics in the emergency department. J Opioid Manag. 11(3):229-236. doi:10.5055/jom.2015.0271.
41. McCarthy DM, Engel KG, Cameron KA. Conversations about analgesics in the emergency department: A qualitative study. Patient Educ Couns. 2016. doi:10.1016/j.pec.2016.01.011.
42. McCarthy DM, Wolf MS, McConnell R, et al. Improving Patient Knowledge and Safe Use of Opioids: A Randomized Controlled Trial. Acad Emerg Med. 2015;22(3):331-339. doi:10.1111/acem.12600.
43. Hinyard LJ, Kreuter MW. Using Narrative Communciation as a Tool for Health Behaviour Change: A Conceptual, Theoretical, and Empirical Overview. Heal Educ Behav. 2007.
44. Kreuter MW, Green MC, Cappella JN, et al. Narrative communication in cancer prevention and control: a framework to guide research and application. Ann Behav Med. 2007;33:221-235. doi:10.1080/08836610701357922.
45. Green MC, Brock TC. The role of transportation in the persuasiveness of public narratives. J Pers Soc Psychol. 2000;79(5):701-721. doi:10.1037/0022-3514.79.5.701.
46. Kreuter MW, Holmes K, Alcaraz K, et al. Comparing narrative and informational videos to increase mammography in low-income African American women. Patient Educ Couns. 2010;81:S6-S14. doi:10.1016/j.pec.2010.09.008.
47. Houston TK, Allison JJ, Sussman M, et al. Culturally appropriate storytelling to improve blood pressure a randomized trial. Ann Intern Med. 2011;154(2):77-84. doi:10.1016/j.ycar.2012.01.099.

48. Petraglia J. Narrative intervention in behavior and public health. J Health Commun. 2007;12(5):493-505. doi:10.1080/10810730701441371.
49. Winterbottom A, Bekker HL, Conner M, Mooney A. Does narrative information bias individuals decision making? A systematic review. Soc Sci Med. 2008;67:2079-2088. doi:10.1016/j.socscimed.2008.09.037.
50. Bekker HL, Winterbottom AE, Butow P, et al. Do personal stories make patient decision aids more effective? A critical review of theory and evidence. BMC Med Inform Decis Mak. 2013;13 Suppl 2:S9. doi:10.1186/1472-6947-13-S2-S9.

References for Study Instruments

1. Stratford PW, Binkley JM, Riddle DL. Development and initial validation of the back pain functional scale. Spine (Phila Pa 1976). 2000;25(16):2095-2102. doi:10.1097/00007632-200008150-00015

2. McNeill JA, Sherwood GD, Starck PL, Thompson CJ. Assessing Clinical Outcomes: Patient Satisfaction with Pain Management. Vol 16.; 1998. doi:10.1016/S0885-3924(98)00034-7.

3. Anderson LA, Dedrick RF. Development of the Trust in Physician scale: a measure to assess interpersonal trust in patient physician relationships. *Psychol Rep*. 1990;67:1091-1100. doi:10.2466/pr0.1990.67.3f.1091.

4. Elwyn, G, Barr PJ, Grande SW, Thompson R, Walsh T, Ozanne Em. Developing CollaboRATE: a fast and frugal patient-reported measure of shared decision making in clinical encounters. Patient Educ Couns. 2013; 93(1):102-7. doi: 10.1016/j.pec.2013.05.009

5. Boscarino J a., Rukstalis M, Hoffman SN, et al. Risk factors for drug dependence among out-patients on opioid therapy in a large US health-care system. Addiction. 2010;105(April 2009):1776-1782. doi:10.1111/j.1360-0443.2010.03052.x

6. GENACIS gender, alcohol, and culture: an international study questionnaire. (2007). Retrieved March 16, 2017 form http://www.genacis.org/questionnaires/core.pdf.

7. ICF Macro, Office on Smoking and Health, Centers for Disease Control and Prevention (CDC). United States National Adult Tobacco Survey 2009-2010. Atlanta, United States: Office on Smoking and Health, Centers for Disease Control and Prevention (CDC).

8. Smith PC, Schmidt SM, Allensworth-Davies D, Saitz R. A Single-Question Screening Test for Drug Use in Primary Care. *Arch Intern Med.* 2010;170(13):1155-1160. doi:10.1001/archinternmed.2010.140

9.Butler, SF, Fernandez, K, Benoit, C, Budman, SH, Jamison, RN. Validation of the revised screener and opioid assessment for patients with pain (SOAPP®-R).J. Pain. 2008; 9 (4):360-372.

10.Chew, DL, Bradley, KA, Boyko, EJ, Brief questions to identify patients with inadequate health literacy. Family Medicine. 2004; 36(8): 588-594.

11. Fagerlin, A., Zikmund-Fisher, B.J., Ubel, P.A., Jankovic, A., Derry, H.A., & Smith, D.M. Measuring numeracy without a math test: Development of the Subjective Numeracy Scale (SNS). *Medical Decision Making*, 2007: 27: 672-680.

12. Sterwart, AL, Hays, RD, Ware, JE. The MOS short-form general health survey. Med. Care. 1988; 26:724-735.

13. Butler, SF, Budman, SH, Fernandez, KC, Houle, B, Benoit, C, et al. Development and validation of the Current Opioid Misuse Measure. PAIN. 2007; 130 (1-2):144-156.

Addendum: Addition of a Qualitative Aim
The overarching goal of Life STORRIED is to assess the comparative effectiveness of probabilistic vs. narrative-enhanced risk communication methods on patient-centered outcomes and opioid use for patients with acutely painful conditions. Narratives are understood to allow patients the ability to assess balanced, evidence-based information on advantages, disadvantages, and consequences of treatment options in a way that is accessible and often less dependent on health literacy or numeracy than other communication methods. Compared to probabilistic strategies, the use of narratives can prompt patients to evaluate relevant information in light of their values, beliefs, and preferences by presenting the information in a way that requires less cognitive effort or bias to attend to more detail. This can help patients understand their unique medical situation by stimulating them to imagine themselves as part of different treatment situations.

In the Life STORRIED trial, the narrative vignettes that were developed using real patients' stories will be evaluated (through the original aims) and compared to probabilistic communication approaches through a series of follow-up surveys assessing risk recall, functional status, opioid use, alignment with provider decisions, and video vignette viewership.

The additional proposed aim builds on compelling observations from the early phases of the Life STORRIED study. *Specifically, during the course of the trial, we have witnessed enrolled subjects have powerful and emotional reactions to the video vignettes*. In the original study design, we did not qualitatively assess how patients with pain perceive these stories, integrate them into their values and preferences, and use them to understand their therapeutic options for pain treatment. To capture this information, which we believe will be vital to building future narrative-based interventions, we seek to add a qualitative component to the Life STORRIED trial. We will conduct telephone interviews with subjects who were enrolled into the narrative arm and, for the sake of comparison, the probabilistic only arm of the study. The aim is described below:

Aim: We will assess how the Life STORRIED subjects --randomized to the narrative and probabilistic only intervention arms -- perceive, share, use, and recall the communication tool and incorporate them into preferences and understanding about pain treatment.
Two of the goals of the narrative and probabilistic interventions are to 1) provide evidence-based risk information in a format that minimizes the cognitive burden of comparing and weighing attributes between treatments, and 2) encourage a conversation and shared decision making process between the patient and provider. The qualitative assessment will seek to understand how subjects perceived, contextualized (through their own experiences), and used (in their own words) the narratives and probabilistic risk tool towards these goals. A secondary goal of the qualitative analysis will be to describe the range of potential explanations that underpin the ultimate findings of the comparative effectiveness trial across the primary and secondary outcomes.

<u>Theoretical Framework</u>: This aim is based on the Ottawa Decision Support Framework (ODSF). The ODSF incorporates patient-level determinants of decisions, decision support interventions, and the impact of interventions on decision quality and health outcomes. This framework is informed by normative and dual process reasoning theories in the fields of cognitive and social psychology.

<u>Protocol</u>: Recruit between 30-40 participants from the Life STORRIED trial who received the probabilistic intervention only (n=10-15) or the narrative and probabilistic intervention (n=25-30) to participate in 20-30 minute telephone interviews. Sampling will be purposive in that we will seek to recruit from trial participants with a variety of clinical conditions (e.g. back pain or kidney stone) from different regions of the country, and from differing age and demographic groups. We will seek to balance these factors while recognizing many trial participants will not be eligible to be contacted if they enrolled prior to 1 year before outreach (as outlined in the original IRB approval). Respondents will be asked to provide informed consent for participation in semi-structured interviews

and for their responses to be merged with data and results from the randomization scheme. They will then participate in individual, semi-structured interviews designed to elicit how they perceive, share, use, and recall the communication tools and incorporate them into their preferences and understanding about pain treatment. After an initial assessment (focusing on recall) each interview will give the participants an opportunity to review the tools that they interacted with at the time of enrollment. Similar to a chart stimulated recall study, participants will be asked to react to these scenarios in an open-ended fashion. Interviews will be audiotaped, transcribed, and entered into NVivo 12.1 for management and analysis. Responses to both phases will be coded using a code book developed by the study team using modified grounded theory. Potential nodes to explore will include: emotional transportation, engagement, recall, cognitive burden of decision making, desire to share with others, role of pain level with engagement of the tool, and substance use stigma. Data analysis will occur at the level of the individual interview participant. Inter-rater reliability for coding will be calculated. Frequency of content analysis codes will be compared across the randomization arms using categorical bivariate analysis such as chi-squared tests. Similarly, results will be explored by demographic and clinical characteristics (back pain vs. kidney stone). Additionally, once the original survey data are unmasked, the qualitative findings will be categorized with the aim of identifying the range of potential perspectives that can demonstrate the underpinnings of the success (or lack of success) in the comparative effectiveness trial.

Informed Consent Process: Study team members will explain the study and Informed Consent process to respondents over the telephone. As the interview will be taking place over the telephone, respondents will be asked to provide verbal consent before participating in the interview.

Compensation: After being consented and participating in the telephone interview, participants will receive $20 on their registered Greenphire ClinCard.

Deliverables: This information is vitally important to the design of future narrative-based health interventions, because of the unique aspect of the challenge of treating pain during an epidemic of opioid use disorder. Currently, little is known about patient preferences for narrative formats, content, and delivery modes—especially when the goals of care include both patient preferences and a desire to limit exposure to harmful (for some) medications. This information will influence design and implementation of future narrative-based interventions as we seek to tailor them to meet the needs of patients and populations with acute and chronic diseases. At the close of the study, this Aim will deliver at least one stand-alone manuscript that explores, in depth, the role of narratives and probabilistic communication for how people understand, contextualize, and use their individual risks when it comes to managing and navigating treatments for acute pain.
