# Supplementary material for: Patient Preference and Risk Assessment in Opioid Prescribing Disparities: A Secondary Analysis of a Randomized Clinical Trial
Source: JAMA Netw Open. 2021 Jul 29;4(7):e2118801. doi: 10.1001/jamanetworkopen.2021.18801 (PMC8322998; doi:10.1001/jamanetworkopen.2021.18801)
Supplement: Supplement 2. — eTable 1. Logistic Regression Models Including Site Fixed Effects eTable 2. Logistic Regression Models Including Interaction Between Race and Study Arm eFigure. CONSORT Flow Diagram [file jamanetwopen-e2118801-s002.pdf]

Supplementary Online Content

Engel-Rebitzer E, Dolan AR, Aronowitz SV, et al. Patient preference and risk assessment in opioid prescribing disparities: a secondary analysis of a randomized clinical trial. *JAMA Netw Open*. 2021;4(7):e2118801. doi:10.1001/jamanetworkopen.2021.18801

eTable 1. Logistic Regression Models Including Site Fixed Effects

eTable 2. Logistic Regression Models Including Interaction Between Race and Study Arm

eFigure. CONSORT Flow Diagram

This supplementary material has been provided by the authors to give readers additional information about their work.

© 2021 Engel-Rebitzer E et al. *JAMA Network Open*.

eTable 1. Logistic Regression Models Including Site Fixed Effects†

	Given an opioid prescription		
	Full Sample (n=968)	*Preferred opioids (n=364)*	*Preferred no opioids (n=602)*
	OR (CI)	*OR* (CI)	*OR* (CI)
**Race**			
White	[reference]	[reference]	[reference]
Black	0.59 (0.36-0.97)	0.50 (0.25-1.00)	0.68 (0.32-1.44)
Other	0.65 (0.39-1.08)	0.65 (0.30-1.44)	0.69 (0.35-1.38)
**Condition**			
Renal colic	[reference]	[reference]	[reference]
Back pain	0.17 (0.12-0.25)	0.19 (0.11-0.34)	0.15 (0.09-0.26)
**Age**	1.00 (0.99-1.02)	1.01 (0.99-1.03)	1.00 (0.98-1.02)
**Gender**			
Female	[reference]	[reference]	[reference]
Male	1.56 (1.11-2.19)	1.23 (0.75-2.03)	1.59 (0.96-2.65)
Other	3.23 (0.24-43.43)	1.98 (0.12-33.62)	
**Education**			
Less than HS	[reference]	[reference]	[reference]
HS or some college	0.61 (0.29-1.26)	0.50 (0.20-1.26)	0.72 (0.19-2.81)
College or more	0.68 (0.31-1.48)	0.34 (0.12-0.97)	1.44 (0.36-5.80)
**Baseline pain level**	1.22 (1.12-1.33)	1.14 (1.01-1.29)	1.24 (1.10-1.39)
**ORT score**	0.97 (0.92-1.02)	0.97 (0.92-1.04)	0.92 (0.84-1.02)
**Narrative arm**	1.07 (0.75-1.53)	1.13 (0.66-1.94)	1.15 (0.68-1.92)
**Site**			
Northwell	[reference]	[reference]	[reference]
Mayo	1.26 (0.78-2.02)	0.67 (0.33-1.36)	1.67 (0.83-3.38)
U Penn	0.46 (0.28-0.75)	0.33 (0.15-0.71)	0.59 (0.29-1.18)
UAB	1.72 (0.91-3.26)	0.83 (0.36-1.92)	3.02 (1.00-9.09)

†Sample size varies for these analyses due to missing demographic, discharge prescription, and treatment preference data.

© 2021 Engel-Rebitzer E et al. *JAMA Network Open*.

eTable 2. Logistic Regression Models Including Interaction Between Race and Study Arm[†]

	Given an opioid prescription		
	Full Sample (n=968)	*Preferred opioids (n=364)*	*Preferred no opioids (n=602)*
	OR (CI)	*OR* (CI)	*OR* (CI)
**Race**			
White	[reference]	[reference]	[reference]
Black	0.73 (0.36-1.45)	0.48 (0.18-1.23)	1.69 (0.58-4.92)
Other	0.83 (0.35-1.93)	0.83 (0.27-2.57)	1.04 (0.27-4.10)
**Condition**			
Renal colic	[reference]	[reference]	[reference]
Back pain	0. 18 (0.12-0.26)	0.20 (0.11-0.35)	0.16 (0.09-0.27)
**Age**	1.00 (0.99-1.02)	1.01 (0.99-1.03)	1.00 (0.99-1.02)
**Gender**			
Female	[reference]	[reference]	[reference]
Male	1.56 (1.11-2.19)	1.23 (0.75-2.02)	1.58 (0.95-2.63)
Other	2.04 (0.13-31.38)	1.48 (0.07-31.74)	
**Education**			
Less than HS	[reference]	[reference]	[reference]
HS or some college	0.59 (0.29-1.21)	0.53 (0.22-1.30)	0.63 (0.16-2.54)
College or more	0.58 (0.27-1.25)	0.37 (0.14-1.02)	1.05 (0.25-4.34)
**Baseline pain level**	1.22 (1.12-1. 32	1.11 (0.98-1.24)	1.24 (1.11-1.40)
**ORT score**	0.96 (0.92-1.01)	0.96 (0.90-1.01)	0.92 (0.83-1.01)
**Experimental arm**	2.10 (1.23-3.60)	1.77 (0.84-3.76)	3.65 (1.54-8.63)
**Narrative arm**	0.87 (0.58-1.31)	0.88 (0.47-1.62)	0.89 (0.49-1.60)
**Interaction term**			
White x experimental arm	[reference]	[reference]	[reference]
Black x experimental arm	0.44 (0.19-1.01)	0.82 (0.26-2.56)	0.13 (0.03-0.51)
Other race x experimental arm	0.61 (0.22-1.66)	0.72 (0.17-3.03)	0.49 (0.10-2.30)

[†]Sample size varies for these analyses due to missing demographic, discharge prescription, and treatment preference data.

© 2021 Engel-Rebitzer E et al. *JAMA Network Open*.

eFigure. CONSORT Flow Diagram

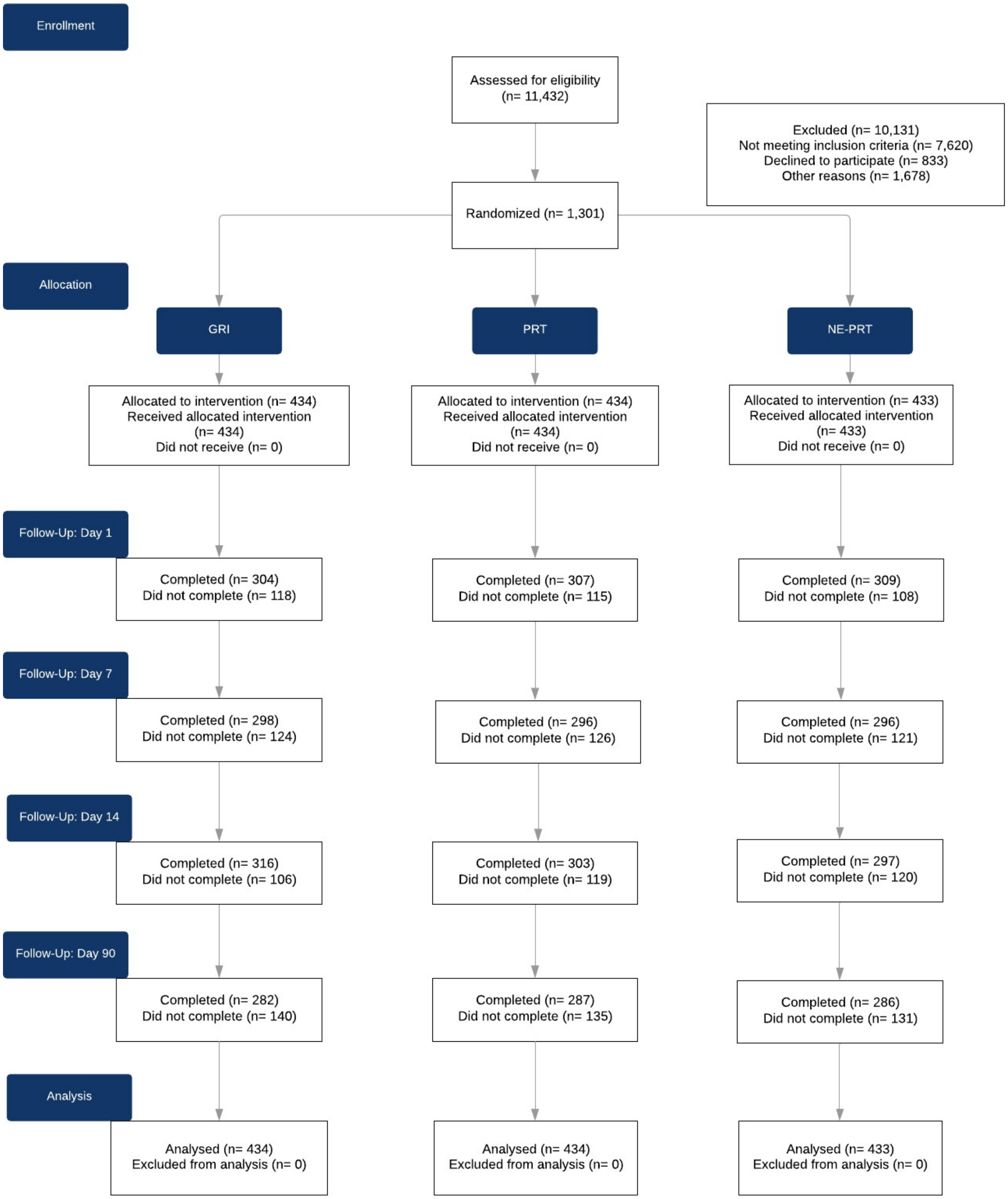

© 2021 Engel-Rebitzer E et al. *JAMA Network Open*.
