# Supplementary material for: Patient Preference and Risk Assessment in Opioid Prescribing Disparities: A Secondary Analysis of a Randomized Clinical Trial
Source: JAMA Netw Open. 2021 Jul 29;4(7):e2118801. doi: 10.1001/jamanetworkopen.2021.18801 (PMC8322998; doi:10.1001/jamanetworkopen.2021.18801)
Supplement: Supplement 3. — Data Sharing Statement [file jamanetwopen-e2118801-s003.pdf]

# Data Sharing Statement

Engel-Rebitzer. Patient Preference and Risk Assessment in Opioid Prescribing Disparities. *JAMA Netw Open*. Published July 29, 2021. doi:10.1001/jamanetworkopen.2021.18801

**Data**
**Data available:** Yes
**Data types:** Deidentified participant data, Data dictionary
**How to access data:** Please contact Abby Dolan at Abby.Dolan@pennmedicine.upenn.edu. All data requests must be approved by The University of Pennsylvania institutional review board.
**When available:** With publication

**Supporting Documents**
**Document types:** None

**Additional Information**
**Who can access the data:** Researchers whose proposed use of the data has been approved (all requests for data must be approved by study staff and by the University of Pennsylvania institutional review board).
**Types of analyses:** For any approved use
**Mechanisms of data availability:** With investigator support after approval of a proposal and with a signed data access agreement. Approval from the University of Pennsylvania IRB is also required.›
